# Anion- and Cation-Specific Response of the Aqueous Conformation of Strong and Weak Polyanionic Brushes

**DOI:** 10.1021/acs.langmuir.5c03349

**Published:** 2025-10-28

**Authors:** Claudia Bertei, Emiliano Bilotti, Julien E. Gautrot

**Affiliations:** † School of Engineering and Materials Science, 4617 Queen Mary University of London, London E1 4ns, United Kingdom; ‡ Department of Aeronautics, Imperial College London, London Sw7 2az, United Kingdom

## Abstract

Polyelectrolyte brushes (PEBs) are surface-tethered polymer chains bearing ionizable groups, whose structure and behavior are highly sensitive to environmental stimuli. When in solution, their response to ions makes them attractive for a variety of applications, including drug delivery, sensing, and smart coatings. In this study, we systematically investigate the specific effect of a wide range of divalent cations and counterions on the swelling behavior of strong and weak anionic PEBs, specifically poly­(3-sulfopropyl methacrylate) (PSPMA) and poly­(acrylic acid) (PAA) brushes. The brushes were synthesized via surface-initiated atom transfer radical polymerization (Si-ATRP) and investigated using in situ ellipsometry and X-ray photoelectron spectroscopy (XPS) to assess swelling behavior and ion retention. Various parameters, including brush thickness, electrolyte chemistry, ionic strength, and pH, were examined. The brushes exhibited significantly different swelling responses depending on the specific ion introduced in solution. Correlations between the observed swelling behavior and key ionic parameters, including hydration enthalpy, ionic radius, pH, and ionic strength, were established. Collectively, these findings offer new insights into ion-specific responsiveness in PEBs and inform the design and optimization of advanced responsive materials for applications in selective ion binding, tunable hydration, and surface functionalization.

## Graphical abstract

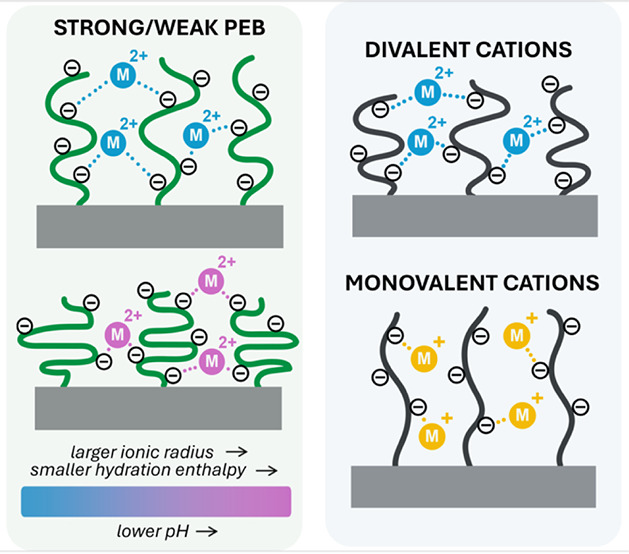

## Introduction

Polyelectrolyte brushes (PEBs) have attracted considerable attention due to their unique properties and versatile potential across various applications, including drug and gene delivery systems,^1–8^ membrane technologies, chromatography, antibacterial coatings, and responsive surfaces.^9,10^ Extensive research has focused on elucidating the responsive properties of PEBs for applications such as sensing, mechanochemistry, and actuation^11,12^ In parallel, several studies have also aimed to understand the ion and molecular interactions that govern the loading of analytes or therapeutics in controlled release systems.^13^ Today, the physicochemical properties of PEBs and their (macro)­molecular interactions with analytes remain key areas of investigation, with an emphasis on enhancing performance and expanding the design of functional interfaces across a growing range of areas.^14^

This is particularly relevant for aqueous applications, where the ionically charged repeating units of PEBs exhibit high sensitivity to environmental conditions such as pH, ionic strength, and type of counterions—factors that significantly influence their conformation and behavior.^15,16^ Depending on the chemistry of the charged residues along the chains, PEBs can be classified as anionic, cationic, or ampholytic (i.e., capable of ionizing into both anions and cations). In addition, they are usually categorized as strong or weak PEBs, according to their ability to display a permanent (i.e., quaternized brushes) or pH-dependent charge, respectively.^17,18^ In the case of strong PEBs, the screening of electrostatic interactions and the swelling of the brush are strongly influenced by the ionic strength of the solution and not the pH. In contrast, for weak PEBs, both ionic strength and pH determine the proportion of charged monomer units, modulating swelling or collapse within the brush.^19,20^

The conformational response of PEBs in complex environments has been receiving significant attention, with the development of theoretical and experimental approaches to study and interpret their specific behavior. In salted environments, distinct swelling responses have been identified for both strong and weak PEBs. Depending on the ionic strength of the aqueous solution, strong PEBs typically exhibit osmotic and salted regimes, whereas weak PEBs can transition through neutral, osmotic, salted, or quasi-neutral states.^21,22^ These swelling regimes reflect the complex interplay of interactions within each system, including brush-ion, brush-solvent, ion-solvent, and self-interactions, which collectively govern brush conformation and response.

The chemistry of the electrolytes in the system plays a critical role in PEB conformation. The specific ion effect, also known as the Hofmeister effect, has often been utilized to understand how different ions modulate molecular interactions and stability in biological and polymeric systems.^23^ The Hofmeister series classifies ions based on their ability to either stabilize (kosmotropes) or destabilize (chaotropes) proteins and polymers in solution.^24,25^ Kosmotropic ions strongly interact with water, maintaining a structured environment that stabilizes biomolecules by reducing their exposure to the solvent. In contrast, chaotropic ions disrupt water structure, leading to protein unfolding and increased solvent exposure of their backbone.^26^ While Hofmeister-related phenomena are generally used as a baseline for interpreting these effects, the principles that underline the specific responses observed in different PEBs are still a matter of investigation and, in some cases, poorly understood.^27^

Ion-responsive behaviors of PEBs have primarily been investigated in the context of polycationic and thermoresponsive systems. For example, poly­(2-methacryloyloxy-ethyl-trimethylammonium chloride) (PMETAC), poly­(dimethylaminoethyl methacrylate) (PDMAEMA), and poly­(2-vinylpyridine) (P2VP) have all been shown to significantly change conformation in response to anion chemistry, with clear correlations between the magnitude of associated conformational changes and the position of these anions within the Hofmeister series. Polycationic brushes were observed to collapse in the presence of chaotropic anions (e.g., perchlorates and thiocyanate), as confirmed through ellipsometry, dynamic light scattering, AFM, and neutron reflectometry.^21,28–30^ These conformational rearrangements were also shown to be associated with significant changes in hydration and viscoelasticity of the brushes, as evidenced by quartz crystal microbalance with dissipation (QCM-D) experiments.^29^ Indeed, combined QCM-D and ellipsometry measurements revealed that weakly basic brushes, such as PDMAEMA, poly­(2-diethylamino)­ethyl methacrylate (poly­(DEAEMA)) and poly­(2-diisopropylamino)­ethyl methacrylate (poly­(DPAEMA)), all exhibit ion-specific swelling and collapse behavior. In contrast, poly­(zwitterionic) brushes were found to display an opposite trend, with brush swelling increasing in the presence of chaotropic thiocyanate ions. This behavior was attributed to the disruption of strong dipole–dipole inter- and intrachain interactions, which would otherwise constrain the conformational freedom of brushes.^31^ Similarly, thermoresponsive polymer brushes (e.g., poly-*N*-isopropylacrylamide (PNIPAM) and ethylene glycol-based brushes) were found to respond strikingly to anions in the Hofmeister series, swelling in the presence of chaotropic species (which increased their lower critical solution temperatures (LCSTs)) and collapsing in the presence of kosmotropic anions (lowering their LCSTs),^32–34^ a behavior that was also observed in PDMAEMA brushes.^35^

In comparison to those of cationic and thermoresponsive brushes, the ion response of polyanionic brushes has received less attention. Poly­(methacrylic acid) (PMAA) brushes were found to swell and become stiffer in the presence of chaotropic cations owing to the formation of effective salt bridges, proposed based on atomistic molecular dynamics simulations.^36^ In another study, poly­(styrenesulfonate) (PSS) brushes revealed cation-specific conformational transitions, where monovalent (Na^+^, Cs^+^) and multivalent (Ca^2+^, La^3+^) cations induced different swelling, collapse, and resolvation behaviors, modulated by solvent composition and charge inversion effects.^37^

Among polyanionic brushes, poly­(3-sulfopropyl methacrylate) (PSPMA) and poly­(acrylic acid) (PAA) brushes have also attracted attention for their unique responses to electrolytes. PSPMA, a strong polyanionic brush, has been studied for its responsive antifouling,^38,39^ biomimetic growth factor capture,^40^ and tribological properties,^30^ for applications in tissue engineering, as well as in microfluidic devices and biosensors. For instance, counterions associated with PSPMA were found to impact the heterogeneous ice nucleation temperature and hydration dynamics of aerosols.^41^ The lubrication properties of PSPMA brushes were found to be modulated by counterion-driven interactions with multivalent ions, associated with their swelling profile and ion response, as studied through combined AFM and QCM-D.^30^ In contrast, Kou et al. found that PSPMA brushes exhibited a more moderate cation specificity, compared to that of polycationic brushes, when exposed to a series of monovalent counterions (Na^+^, K^+^, Cs^+^), with variations in brush hydration largely attributed to differences in bound molar mass rather than ion-specific interactions.^42^ While ion valency and coordination critically influence brush–ion interactions, distinct behaviors have also been achieved by introducing functional counterions. For example, azobenzene-based photoresponsive counterions enabled the reversible modulation of brush hydration and conformation upon UV and visible light exposure. The photoresponse could be finely tuned by varying the ratio of azobenzene to potassium ions, highlighting the ability of counterions to modulate external stimulus responsiveness in strong PEBs.^43^

PAA brushes, on the other hand, are weak polyanionic systems and have been explored in drug delivery applications due to their ability to release therapeutics based on local pH conditions. For example, their use in the controlled release of chemotherapy drugs in acidic tumor microenvironments enabled the reduction of treatment toxicity.^44^ Additionally, PAA brushes have been applied in metal ion remediation, where their ability to coordinate with cations is leveraged for selective ion uptake.^45^ Moreover, recent ellipsometry and FTIR studies revealed that multivalent cations (e.g., Ca^2+^, Ce^3+^) can induce significant changes in PAA brush conformation by shifting the p*K*_a_ and dissociation behavior of carboxylic groups, leading to swelling even under acidic conditions through bridging coordination.^46^ These interactions are not limited to pH or metal complexation alone: the ionic strength also plays a critical role. For example, Hollmann et al. demonstrated that protein binding to PAA brushes is strongly modulated by salt concentration: at low ionic strength, both positively and negatively charged proteins were readily adsorbed, while at higher ionic strength, adsorption was largely suppressed, highlighting their potential for selective protein immobilization.^47^ Understanding the interactions of both PSPMA and PAA brushes with different types of electrolytes is therefore particularly important, as ion-induced conformational changes give rise to diverse behaviors capable of unlocking a variety of functionalities, from controlled drug release to surface adhesion.

In this study, we systematically investigated and compared the ion response of weak and strong anionic polyelectrolyte brushes. Specifically, the swelling behavior and ion retention of PSPMA and PAA brushes were evaluated in the presence of a broad range of biologically and pharmacologically relevant divalent cations, including Mg^2+^, Ca^2+^, Mn^2+^, Fe^2+^, Ni^2+^, Cu^2+^, Zn^2+^, Sr^2+^, and Sn^2+^.^48–50^ A small set of anions, namely ClO_4_^–^, F^–^, NO_3_^–^, and Cl^–^, was also investigated for comparison. For this purpose, ellipsometry and XPS were employed to characterize the PEBs’ response to these species across a range of environmental conditions, which enabled the determination and interpretation of their interactions and associated structural changes.

## Experimental Section

### Materials

For the polymerization of the polymer brushes, anhydrous toluene (99.8%), triethylamine (99%), methanol (99.9%), ethanol (99.5%), 3-sulfopropyl methacrylate potassium salt (*M*_n_ = 246.32; 98%), *tert*-butyl acrylate (tBA; *M*_n_ = 128.17, 98%), copper­(II) chloride (CuCl_2_; 97%), copper­(I) chloride (CuCl; 99.995%), 2,2′-bipyridyl (bpy; 99%), *N,N,N’,N”,N”*-pentamethyldiethylenetriamine (PMDETA; 99%), copper­(I) bromide (CuBr; 99.995%), copper­(II) bromide (CuBr_2_; 99.999%), dichloromethane (DCM; 99.8%), and trifluoroacetic acid (TFA; 99%) were purchased from Sigma-Aldrich. The ATRP initiator, 3-trimethoxysilyl-propyl 2-bromo-2-methylpropionate, was purchased from Gelest and stored at −20 °C. CuCl and CuBr were kept under vacuum until used. Ethylenediaminetetraacetic acid tetrasodium salt hydrate (EDTA; 98%) was purchased from Fluka and used to make a 10 mM solution. Silicon wafers (100 mm diameter, ⟨100⟩ orientation, one side polished) were purchased from Pi-Kem Limited and cleaned in a Henniker Plasma Cleanser (HPT-200) for 5 min in air at 100% power.

For the preparation of aqueous solutions, calcium chloride (CaCl_2_; 99.99%), strontium chloride hexahydrate (SrCl_2_·6H_2_O; 99%), zinc chloride (ZnCl_2_; 98%), manganese chloride tetrahydrate (MnCl_2_·4H_2_O; 99%), nickel chloride (NiCl_2_; 99.99%), magnesium chloride (MgCl_2_; 98%), iron­(II) chloride tetrahydrate (FeCl_2_·4H_2_O; 99.0%), stannous chloride (SnCl_2_; 99.99%), sodium chloride (NaCl; 99.0%), sodium fluoride (NaF; 99%), sodium perchlorate (NaClO_4_; 98%), and sodium nitrate (NaNO_3_; 99%) were also purchased from Sigma-Aldrich.

### Preparation of Dry PSPMA and PAA Brushes

Silane initiator deposition was carried out following a protocol previously reported.^51^ Pieces of plasma-oxidized silicon wafers were cut to the desired size and immersed in a Petri dish containing anhydrous toluene (30 mL), anhydrous triethylamine (50 μL), and the silane initiator (30 μL); covered with aluminum foil; and left at room temperature overnight. The wafers were then washed with ethanol and dried under a stream of nitrogen.

PAA brushes were prepared by first polymerizing poly­(*tert*-butyl acrylate) (P*t*BA), which was selected for its excellent polymerization control,^52^ followed by deprotection of the *tert*-butyl groups. To remove the inhibitor used to stabilize *tert*-butyl acrylate (monomethyl ether hydroquinone), the monomer was purified using a basic alumina column and subsequently distilled under vacuum (78 mbar) at 61–63 °C. The distilled reagent (30 mL, 205 mmol) was added to a solution of PMDETA (122 μL, 584 μmol), CuBr_2_ (3 mg, 13 μmol), CuBr (6.5 mg, 45 μmol), and acetone (16 mL), which was previously degassed using argon and heated to 60 °C. A 1 × 1 cm piece of plasma-treated silicon wafer was inserted in a sealed flask that was previously degassed via three cycles of high vacuum and argon gas. Polymerization was then carried out at 60 °C under an inert atmosphere. To stop the polymerization, the samples were immersed in acetone, rinsed in ethanol and deionized water, and then dried under a stream of nitrogen. To deprotect the P*t*BA brushes, the samples were immersed in a solution of DCM/TFA (10:1 v/v) and left overnight at room temperature. The next day, the PAA brush samples were washed in ethanol and deionized water and dried under a stream of nitrogen.

For the polymerization of PSPMA brushes, 3-sulfopropyl methacrylate potassium salt (8.65 g, 35.12 mmol), CuCl_2_ (0.0255 g, 190 μmol), CuCl (0.0825 g, 833 μmol), and bpy (0.3255 g, 2.08 mmol) were dissolved in 15 mL of various solvent mixtures containing different methanol/water ratios and degassed with argon gas. Plasma-treated silicon wafer pieces of 1 × 1 cm or 2.5 × 2.5 cm were inserted in clean reaction tubes and degassed via three cycles of high vacuum and argon gas refilling, before the injection of the reaction mixture. To stop the polymerization, the samples were immersed in deionized water, rinsed with ethanol and deionized water, and then dried under a stream of nitrogen.

### Removal of Cu Catalyst Residues

To remove copper complex residues after polymer brush formation, the samples were washed with a 10 mM aqueous solution of EDTA. The pH of the solution was measured with a Mettler Toledo FiveGo F2 pH meter, which reported a pH value of 9.30 at room temperature. The planar samples that underwent the EDTA step were dipped in the solution for 30 seconds, then rinsed with abundant deionized water and ethanol, and dried under a stream of nitrogen.

### Ellipsometry and In Situ Ellipsometry

Dry and in situ swollen brush thicknesses were measured using an α-SE ellipsometer from Woollam Co. Inc., Ellipsometry Solutions. ϕ and Δ were measured over a wavelength range of 400–900 nm at a fixed incidence angle of 70°. For in situ measurements, which required the samples to be submerged in various solutions, an open chamber with quartz windows oriented normally to the beam direction was used. The polymerization process was also monitored using ellipsometry to assess oxide, silane initiators, and PB layer formation. For every solution tested, the optical parameters of the medium were acquired beforehand (Table S2) using a reference silicon chip with a known oxide thickness (25 nm). Each measurement was fitted with a Cauchy model that enabled the determination of the optical constants (including the refractive index) of the solutions used. For in situ ellipsometry, each sample was allowed to equilibrate after immersion in the chamber containing the solution for 1 min prior to data acquisition. After this time, further evolution of the ellipsometric properties was not observed. Brush stability was confirmed by comparing dry thicknesses at the beginning and end of each series of measurements. All measurements were taken in triplicate and displayed mean squared errors (MSEs) < 5. A Cauchy model was used to fit ellipsometry raw data. A one-way ANOVA test with Tukey’s post hoc analysis was used to determine the statistical significance of the brushes’ swelling response and can be found in the Supporting Information.

### X-ray Photoelectron Spectroscopy

XPS was carried out using a Thermo Scientific K-Alpha X-ray XPS System equipped with a monochromatic AlKα source, operating under ultrahigh vacuum conditions (109 mbar). Survey spectra were collected from 1300 to 0 eV, and high-resolution XPS spectra were acquired for C, O, N, S, K, Na, and a variety of metals. Ion retention was assessed through XPS after silicon chips with PSPMA, and PAA brushes were first treated with EDTA solution and then incubated in 5 mL aqueous solutions of the desired salt for 10 min. The samples were then rinsed with abundant deionized water and ethanol and dried under a stream of nitrogen before being positioned on the XPS sample holder. All measurements were taken in triplicates.

## Results and Discussion

To investigate the behavior of strong and weak anionic PEBs under varying conditions, PAA and PSPMA brushes were synthesized on silicon substrates via surface-initiated atom transfer radical polymerization (SI-ATRP). The synthesis of PAA and PSPMA brushes on silicon wafers was adapted from previously reported protocols (Figure 1A,B).^51^ The growth kinetics of PSPMA brushes were varied to obtain different brush thicknesses. This was achieved by changing the ratio of methanol/deionized water in the reaction mixture and varying the polymerization time (Table S1). PSPMA samples displayed a characteristic color ranging from silver-orange, for relatively thin brushes (40 nm), to light/intense blue, for thicker brushes (Figure 1C–E). This color change is a result of different factors, including thickness-dependent light refraction and the presence of optically active copper complexes retained from the ATRP process (prior to EDTA washing).

**Figure 1:**
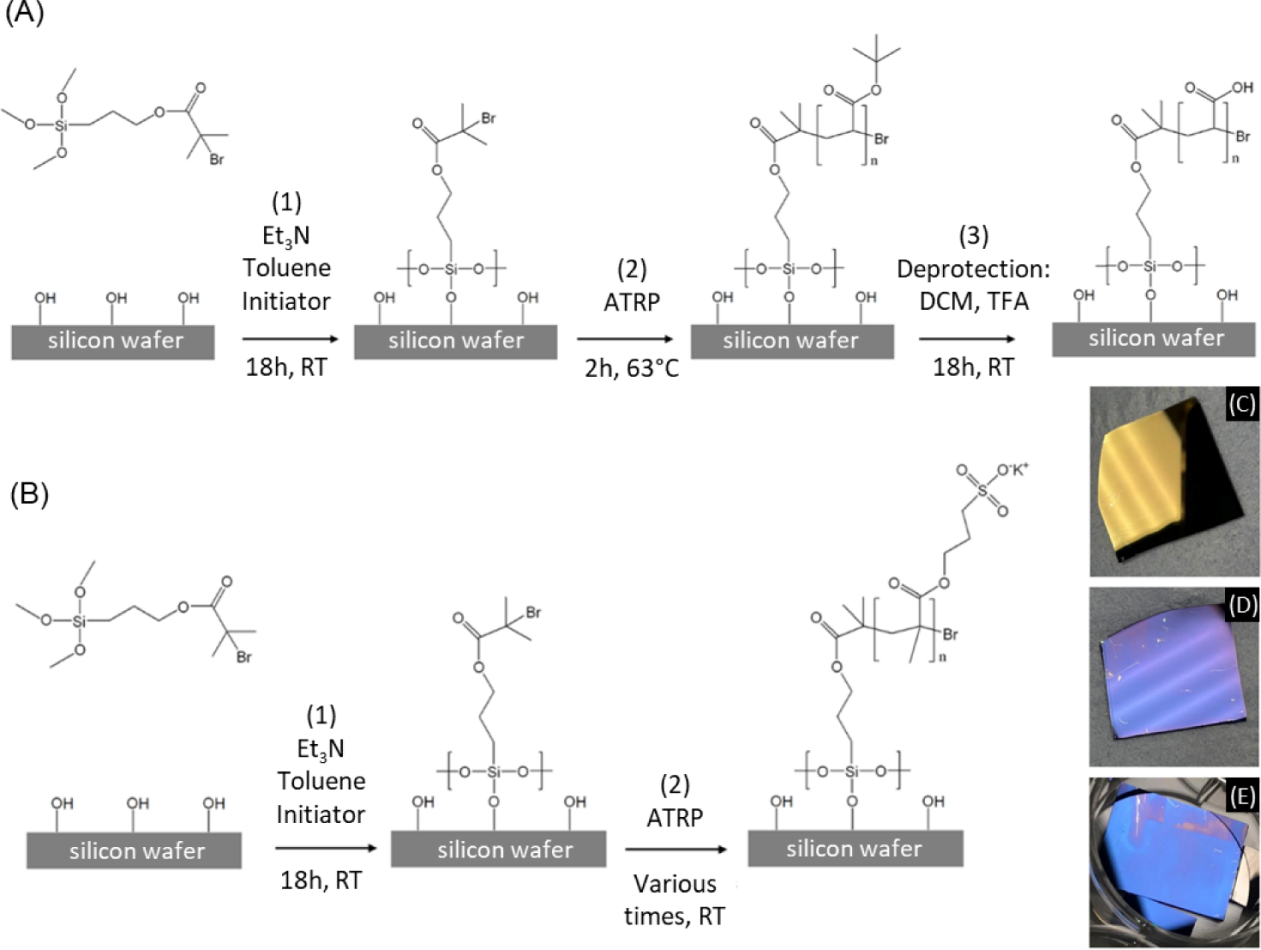
Schematic of the multistep polymerization process of PAA brushes: (1) initiator deposition, (2) Si-ATRP of tBA, and (3) deprotection of P*t*BA brushes to obtain PAA brushes (A). Schematic of the polymerization process of PSPMA brushes: (1) initiator deposition and (2) Si-ATRP of 3-sulfopropyl methacrylate (B). Pictures of pristine PSPMA brushes with thicknesses of ∼40 nm (C), ∼65 nm (D), and ∼100 nm (E).

PAA brushes revealed an approximate ∼54% reduction in dry brush thickness postincubation in DCM/TFA solutions, which was attributed to the removal of *tert*-butyl groups. The steric hindrance of the *tert*-butyl groups was expected to influence the brush density, affecting the number of chains growing per nm^2^. Previous experimental reports showed that grafting density significantly affects the swelling behavior of charged brushes, a phenomenon fully supported by a broad range of studies.^53–55^ For instance, a study on low-density PDMAEMA brushes showed that the brushes exhibited greater responsiveness to pH changes compared to those with higher grafting density, which displayed pH-independent swelling.^56^ In this context, it was hypothesized that the PAA brushes, obtained via deprotection of P*t*BA, possessed a lower grafting density than the directly polymerized PSPMA brushes.^55,57^ Although grafting densities were not directly measured in this study, they were approximated from polymerization conditions and literature comparisons to be ∼0.5 chain/nm^–2^ for PSPMA and ∼0.4 chain/nm^– 2^ for P*t*BA brushes.^52^ Such differences in brush density are known to significantly affect swelling, with sparser brushes adopting a more coiled conformation with larger interchain spacing, which allows easier solvent penetration but results in reduced chain stretching and weaker steric or electrostatic repulsion.^55,58^ Additional factors such as differences in side chain size, charge, and hydration state were also accounted for when analyzing swelling responses of the two brushes.^59,60^

XPS spectra of pristine PSPMA brushes confirmed their surface chemical composition (Figure 2A, C), showing two characteristic sulfur peaks at ∼168 eV and ∼231 eV, attributed to S 2p and S 2s signals, respectively. Additionally, strong copper and nitrogen peaks were observed, indicating the retention of a considerable amount of the copper-bipyridine catalyst from the ATRP process, as reported in another study.^61^

**Figure 2:**
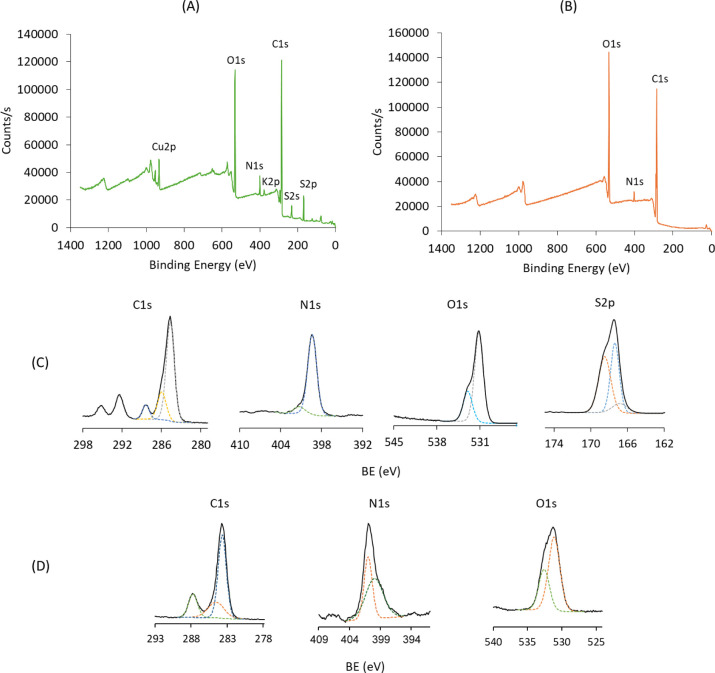
Wide scan XPS spectrum (A) and high-resolution spectra of carbon, oxygen, nitrogen, sulfur, and copper (C) of a representative pristine PSPMA brush. Wide scan XPS spectrum (B) and high-resolution spectra of carbon, oxygen, and nitrogen (D) of a representative pristine PAA brush. Dashed colored lines represent individual fitted components corresponding to distinct chemical environments; the black solid line corresponds to the data.

XPS analysis of pristine PAA brushes was also carried out to confirm surface chemical composition (Figure 2B, D), revealing distinct carbon and oxygen peaks attributed to the PAA polymer chains, in agreement with the literature.^62^ Notably, no bromine signal within the 66–72 eV range (Br 3d) was detected, while the nitrogen peak at ∼401 eV was attributed to PMEDTA residues from the ATRP process. Interestingly, there were no discernible copper-related peaks in the wide scan of the PAA samples. This suggested that the deprotection step, involving overnight incubation in TFA/DCM, may have contributed to the partial removal of catalyst complex residues and reduced their detectability.

Copper-catalyst residues were anticipated to influence the swelling behavior of both PSPMA and PAA brushes. However, it was observed that neither briefly washing the pristine samples nor incubating them for 10 min in deionized water could effectively remove Cu-bpy from PSPMA brushes, even at the most superficial level. In fact, after incubation in deionized water, the nitrogen peak at 399.84 eV from the XPS spectra remains clearly visible, along with all copper peaks (Figure 3A). The only noticeable change postincubation is the disappearance of potassium peaks, previously observed at approximately ∼377 and ∼293 eV in pristine samples. A solution of EDTA was then utilized to attempt chelating and removing the residual copper complexes. After immersion of the samples for 30–60 s in the solution, successful removal was confirmed via XPS, as shown in the spectra of PSPMA samples pre- and post-EDTA treatment in Figure 3A. Following this treatment, two new peaks appeared at 1071.2 and 497.1 eV, corresponding to Na 1s and Na KL1, respectively. These features indicate the replacement of the original potassium counterions associated with the sulfonate groups by sodium ions from the EDTA solution.

**Figure 3:**
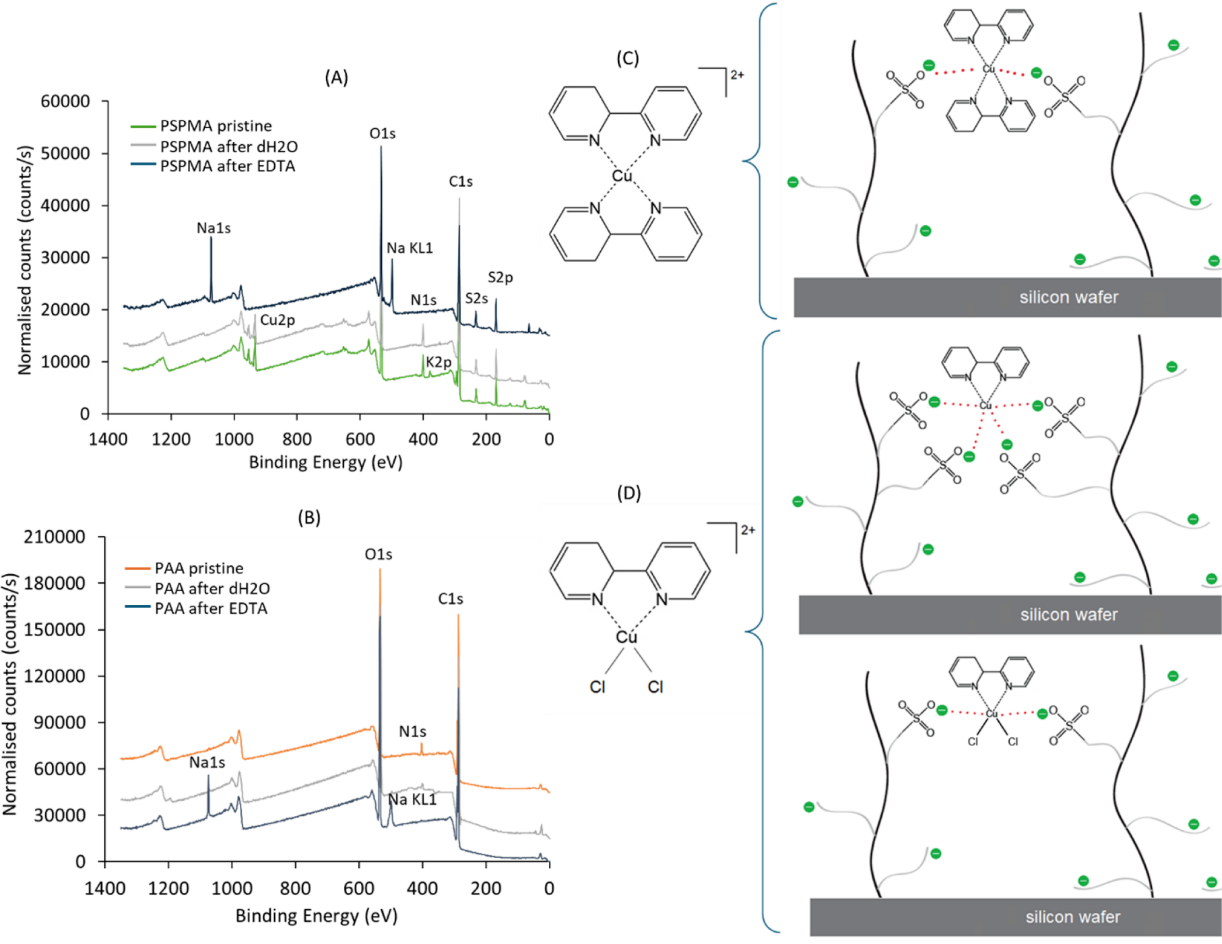
XPS spectrum comparison of pristine PSPMA brush (blue), PSPMA brush incubated in dH_2_O (orange), and PSPMA after incubation in 10 mM EDTA solution (gray) (A). XPS spectrum comparison of pristine PAA brush (orange), PAA brush incubated in dH_2_O (gray), and PAA after incubation in 10 mM EDTA solution (dark blue) (B). Representation of the possible complexes between copper and 2,2-bipyridine (C,D) and their hypothesized interaction mechanisms with PSPMA brushes.

From the atomic % of pristine PSPMA brushes (see Table S4), it is possible to observe that the ratio between Cu 2p and N 1s is ∼1:3, suggesting that copper might have been taken up in the brushes as a mixture of Cu-bpy_2_ and Cu-bpy. It was, therefore, hypothesized that the copper complex could be strongly retained within the brushes because of electrostatic interactions with the sulfonate groups of PSPMA chains. This may have been enabled by the ability of Cu^2+^ cations to expand their coordination sphere, allowing multiple interactions, both with bpy moieties and neighboring polymer chains. Studies show that the coordination number in copper­(II) complexes can vary significantly: four-, five-, and six-coordinate complexes are commonly reported, although the most usual remain hexadentate species.^63,64^ As a result, proposed interactions between the residual Cu-bpy_2_/Cu-bpy complexes and PSPMA brushes are shown in Figure 3C,D.

The removal of the Cu-bpy_2_/Cu-bpy complex had repercussions not only on the surface chemistry of the brushes but also on their swelling behavior. It was reasonable to speculate that charged multidentate or multivalent species, such as the complexes observed within PSPMA brushes, could potentially create physical “crosslinks” between polymer chains, restricting their conformational freedom and swelling. This phenomenon was indeed observed through in situ ellipsometry measurements of PSPMA brushes in deionized water, conducted before and after EDTA treatment (see Figure S19). The treatment led to a clear increase in brush swelling. Specifically, brushes with an initial dry thickness of approximately 31 nm exhibited an ∼71% greater swelling in deionized water post-EDTA treatment compared to untreated brushes. On the other hand, residue removal led to a more modest swelling increase of ∼17% and ∼8% in 48 nm and 64 nm brushes, respectively.

This observation aligns with expectations as thinner PSPMA brushes were anticipated to be more affected by complex removal than thicker ones. In thicker brushes, incomplete removal may have resulted from the limited diffusion of EDTA into the deeper regions of the brush. This limitation is likely due to the high density of the polymer brush, preventing EDTA molecules from chelating complexes deeply embedded within the chains, even after prolonged incubation. Additionally, incomplete removal in thicker brushes may also be linked to their higher initial complex loading as longer polymer chains are likely to trap a larger number of molecules. Consequently, the incubation time may not have been sufficient for EDTA to remove all the complex residues.^65,66^ It could also be proposed that catalyst infiltration was not comparatively as deep in thicker brushes, again owing to the more crowded environment associated with these brushes and the increased entropic penalty. Nonetheless, extended EDTA treatments were deliberately avoided to minimize the risk of chain detachment, particularly as both PSPMA and PAA brushes tethered to silicon substrates have been shown to degraft over time under aqueous or high ionic strength conditions due to swelling-induced tension at the substrate interface.^67–69^ To mitigate this, all brushes were used within 3 months of preparation and stored under inert conditions between incubations. No visible degradation or loss of brush thickness was observed during the timescale of the experiments. Moreover, the selected EDTA treatment duration was sufficient to achieve effective copper removal (confirmed by XPS) and resulted in significant and reproducible changes in dry and swollen brush behavior across all tested samples. For long-term or high salt content applications, more robust anchoring strategies may be required to enhance brush stability and prevent substrate etching.^67^

Similarly to what was observed for PSPMA, residual PMDETA from P*t*BA polymerization was also detected and removed through EDTA treatment of PAA brushes. Unlike PSPMA brushes, for which swelling behavior was examined across multiple thicknesses, comparable variation could not be achieved with PAA due to limitations in brush growth. Therefore, only PAA brushes with the highest achieved thickness (∼30 nm) were included in the study. The XPS wide scan comparison (Figure 3B) shows that nitrogen peaks at 401.15 eV, corresponding to PMDETA, are clearly visible both before and after incubation in deionized water. Instead, following incubation in EDTA solution, the disappearance of the nitrogen peak previously attributed to PMDETA confirms the successful removal of the residual catalyst. Two hypotheses may explain this observation: either copper was always present but remained undetected in the initial XPS analysis, or the retention of PMDETA alone may have resulted from physical entrapment within the polymer network, possibly stabilized by weak polar interactions. In addition, the appearance of sodium peaks at 1071.78 and 497.14 eV post-EDTA treatment indicates that the high pH of the solution (measured as 9.3) likely ionized a significant proportion of COOH groups on the PAA chains, exchanging protons with Na counterions (Table S3). This is in line with previous studies, showing that PAA brushes are considered fully ionized at pH levels above 9.^70^

Dry and in situ ellipsometry measurements were also conducted on PAA brushes to evaluate the impact of EDTA treatment on their swelling and collapse behavior (Figure S19). The effect of the EDTA treatment on the dry thickness of PAA brushes was examined, revealing only a 3% reduction after incubation. Such a minimal difference could be attributed to a few reasons, such as a potentially lower absorption of PMDETA relative to bpy ligands in PSPMA brushes. In fact, the absence of charged and hydrogen-bonding functionalities in the pristine P*t*BA brushes may have limited PMDETA retention, while partial removal of the residual complex likely occurred during the TFA/DCM deprotection step.

In situ ellipsometry was then utilized to assess how different types of cations in solutions influenced PSPMA and PAA brush swelling. For the solutions used in these experiments, chloride was kept as the counterion for all solubilized salts, each at a concentration of 10 mM. The impact of cation chemistry on brush swelling was evaluated by calculating swelling ratios as swollen height/dry height, utilizing dry thickness values after EDTA treatment, unless specified otherwise. To ensure comparability across salt exposures and avoid constraint effects from retained cations (Figure S20), an EDTA washing step was introduced after each incubation. XPS later confirmed that, as with the copper catalyst, divalent cations remained trapped despite rinsing with water, which confirmed that this step was necessary to prevent swelling artifacts.

In parallel with the cation study, the potential effect of the thickness of PSPMA brushes (and therefore their molecular weight) on their swelling behavior was also examined, as this remains an area for which conflicting evidence is reported in the literature. While some reports suggest that swelling increases with brush thickness, others have found swelling to be independent of molecular weight,^54,71^ or even to decrease with increasing thickness.^55,72^ To the best of our knowledge, the effect of PSPMA molecular weight on brush swelling in various electrolyte solutions has not been previously reported and was therefore investigated in this study.

Swelling ratios in each salt solution, for three PSPMA brushes with different dry thicknesses, were calculated from in situ ellipsometry measurements and are plotted in Figure 4A. It was observed that the swelling ratio of the brushes in deionized water was markedly higher than in divalent cation solutions. This was attributed to an osmotic regime, with the reduction likely arising from a combination of electrostatic screening and ion-bridging.^22,73^ It was also observed that for thicker brushes, the swelling ratio was overall lower, in agreement with the behavior of other polymers such as PAA, PSS, and PNIPAM previously investigated.^56,57,74^ Factors such as residual catalyst impurities, dispersity of the polymer chains associated with high thicknesses, and possible gradual chain desorption could play a role in the observed behaviors. The potential dispersity of brushes was explored through the analysis of the in situ ellipsometry data via the incorporation of a roughness parameter when fitting the measurements (as suggested by the software). Although ATRP typically yields relatively monodisperse polymers in moderately dilute solution polymerizations, dispersities can vary in brushes with moderate to high grafting densities and relatively high thicknesses.^66,75,76^ The observed dispersity was hypothesized to arise from a reduction in the availability of active polymerization sites due to termination and steric crowding during chain propagation, leading to increasing inhomogeneities over time. This effect may have been further exacerbated by the inherently higher rates of bimolecular chain termination in surface-initiated polymerizations compared to those in solution, which are known to further contribute to molecular weight dispersity.^77^

**Figure 4:**
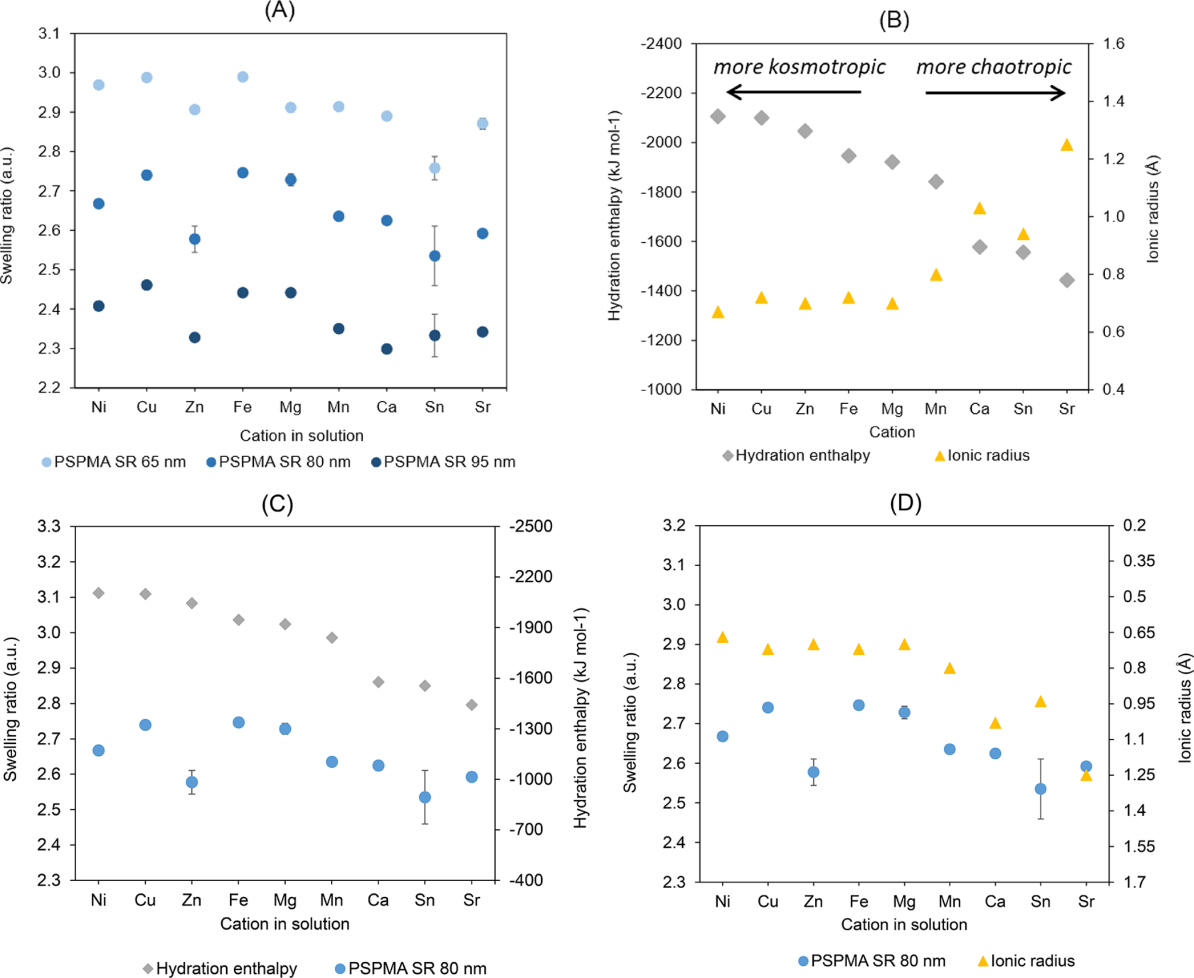
Swelling ratios of PSPMA brush samples with varying initial dry thicknesses measured via in situ ellipsometry in 10 mM chloride salt solutions containing nine different cations (A). Literature values for hydration enthalpies and ionic radii of the cations (shown in order of decreasing absolute value of hydration enthalpy) highlight their opposing trends (B). Graphs compare the swelling ratios for PSPMA brushes with an initial dry thickness of 80 nm (as a representative sample) versus the hydration enthalpies (C) and ionic radii (D) of the corresponding cations in solution. For comparison, the average swelling ratio of PSPMA brushes in deionized water was 2.87, 4.03, and 4.34 for dry thicknesses of 95 nm, 80 nm, and 65 nm, respectively (these data points are not shown in the graphs for clarity).

It was observed that Sn^2+^, Zn^2+^, Sr^2+^, Ca^2+^, and Mn^2+^ induced weaker swelling of PSPMA brushes compared to Ni^2+^, Mg^2+^, Cu^2+^, and Fe^2+^. To interpret these differences, several parameters were considered besides the Hofmeister effect, including ion size and hydration mechanisms.^42,76^ Specifically, given the known influence of ion–water interactions and electrostatic effects on polyelectrolyte behavior, it was considered appropriate to correlate the evolution of swelling ratios with experimental values of hydration enthalpy^78^ and ionic radii in solution^79^ reported in the literature.

Experimental hydration enthalpies of the cations used in this study were compared to their ionic radii (Figure 4B), confirming that smaller ions generally exhibit larger hydration enthalpies due to their higher charge density and stronger electrostatic interactions with water molecules. Consistent with this trend, increased swelling of PSPMA brushes was correlated with increasing hydration enthalpies and decreasing radii overall (Figure 4C,D). Building on these observations, we propose that in the osmotic regime (with all electrolytes at 10 mM), the hydrodynamic radius of the ions and their effective charge density (as reflected by hydration enthalpy and ionic radius) both play key roles in determining and predicting brush swelling. These results are in good agreement with previous reports, indicating that ions can promote salt-bridge formation, physically cross-linking polyelectrolyte chains, and thereby constraining their extension.^80^ In our system, we find that predominantly chaotropic ions, with lower hydration enthalpies and larger ionic radii, are more effective at promoting bridging effects, which constrain PSPMA chains, reduce their stretching, and consequently limit swelling. The observed behavior does not follow the Irving–Williams series, typically used to describe relative stabilities of divalent transition-metal complexes, which is to be expected considering the nature of the interactions studied in the present work. Brush response was observed to be governed by ion–water interactions and effective charge density rather than complex stability. Moreover, variations in such behavior depending on the thickness of PSPMA brushes may be due to alterations of the brush morphology in the *x*–*y* plane, such as the formation of clusters, which result in increased roughness (e.g., for Mg^2+^; see ellipsometry fitting results comparing two different models in Figure S21). It should also be noted that deviations from expected trends for solutions containing Zn^2+^ and Sn^2+^ could be associated with the formation of zinc oxychloride and tin hydroxychloride salts (as suggested by optical microscopy analysis; see Supporting Information).^81,82^

The swollen thickness of PAA brushes was also monitored using in situ ellipsometry, and the resulting swelling ratios in the presence of the different divalent cations were quantified and are plotted in Figure 5A. The effects of the specific cations were found to be significantly different only when comparing Sn^2+^, Zn^2+^, and Mn^2+^ response to Fe^2+^, Sr^2+^, and Mg^2+^, with the first group showing to be more effective at inducing the collapse of PAA brushes (i.e., chaotropic behavior) and the second causing the brushes to swell more (i.e., kosmotropic behavior). No significant difference was observed among Mn^2+^, Zn^2+^, Ca^2+^, Cu^2+^, and Ni^2+^ responses. As reported for PSPMA brushes, evidence of physical “cross-linking” and salt bridging caused by divalent cations was also observed for PAA brushes. However, significant deviation compared to the expected effect was found in some cases, in particular Sr^2+^ and Mg^2+^ (Figure 5B,C), which had a more pronounced effect on swelling than predicted (particularly considering their low to moderate hydration enthalpies). To interpret these findings, changes in solution pH were taken into account. pH plays a critical role in determining the ionization degree of weak PEBs, which in turn influences their conformation and their ability to interact with surrounding species. The pH shifts observed in electrolyte solutions were interpreted to arise primarily from cation-specific modulation of water ionization, rather than from direct proton exchange with the brushes, given the very low equivalent concentration of carboxylates associated with brushes on flat substrates (these changes occurred in the absence of brushes too). Indeed, we observed that higher pH values (particularly in SrCl_2_ and MgCl_2_ solutions) led to increased chain stretching, likely due to enhanced charge repulsion, which resulted in greater swelling of the brush (Figure 5D). These findings suggest that the solution conformation of PAA brushes is governed by a combination of pH (modulated by electrolytes present), ionic radius, and hydration enthalpy. The overall collapse of PAA brushes upon exposure to divalent cations was also considerably more pronounced than that observed for PSPMA brushes (as indicated by the larger decrease in swelling relative to deionized water). This stronger collapse is likely due to the higher hydration energy of carboxylate groups compared to sulfonates,^78^ which makes them more susceptible to dehydration and ion-induced contraction. The displacement of associated water molecules upon binding of divalent cations may therefore have contributed to the pronounced reduction in brush swelling

**Figure 5:**
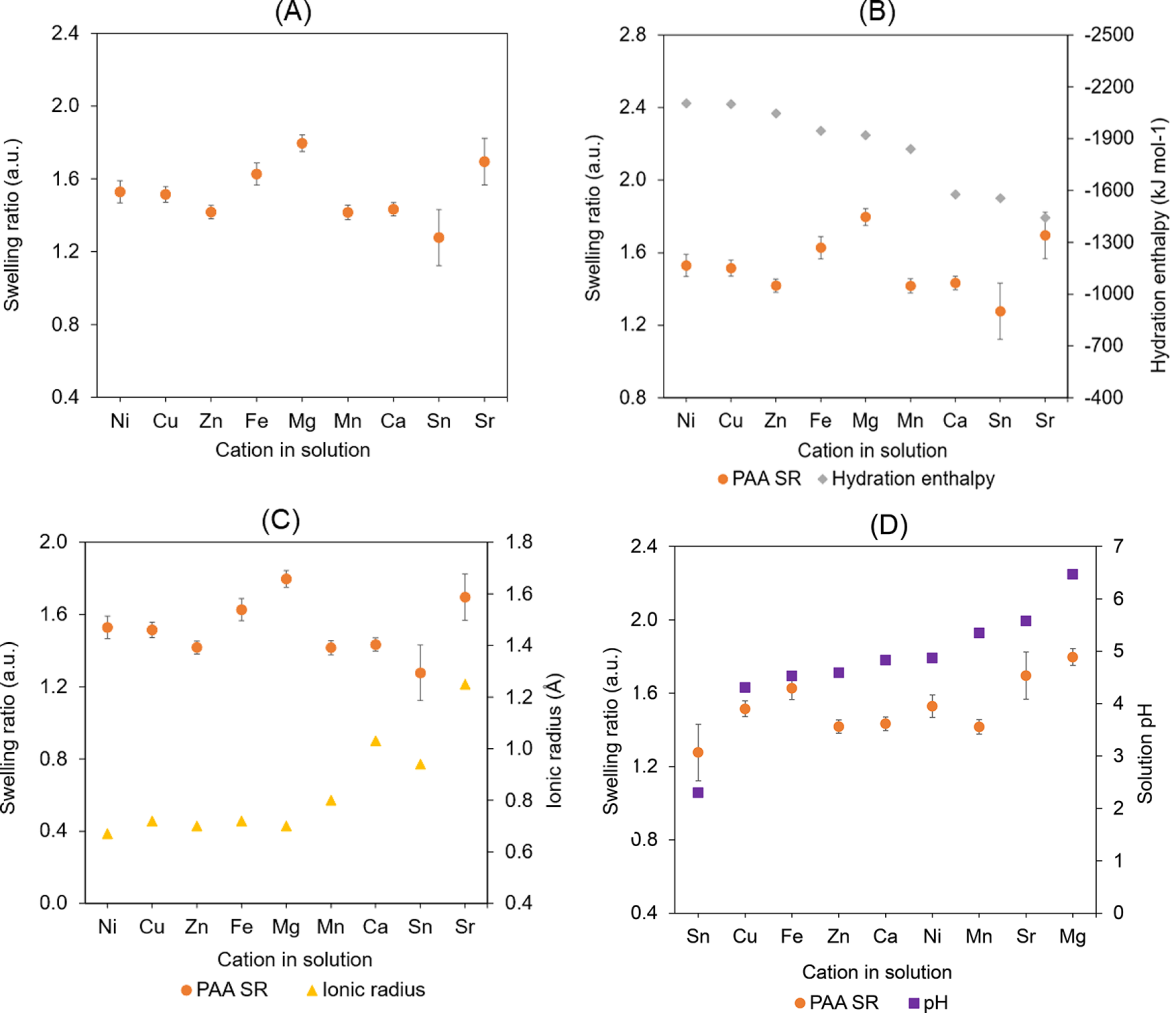
Swelling ratios of PAA brushes tested via in situ ellipsometry in 10 mM chloride salt solutions containing 9 different cations (A). Graphs comparing the trends of the average values of swelling ratios of PAA brushes with the hydration enthalpies (B), ionic radii of the corresponding cations in solution (C), and the pH of the solution containing the corresponding cation, measured with a pH meter (D). For comparison, the average swelling ratio of PAA brushes in deionized water was 3.56 (the data point is not shown in the graphs for clarity).

The absorption and retention of divalent cations within PSPMA and PAA brushes after incubation in 10 mM solutions of the respective salts were confirmed and analyzed via XPS (Figures S10–S18).

The high-resolution spectra of each cation displayed in Figure 6 demonstrate that all ions were clearly detected in both brushes, despite the thorough washing of each sample with deionized water and ethanol prior to measurements.

**Figure 6:**
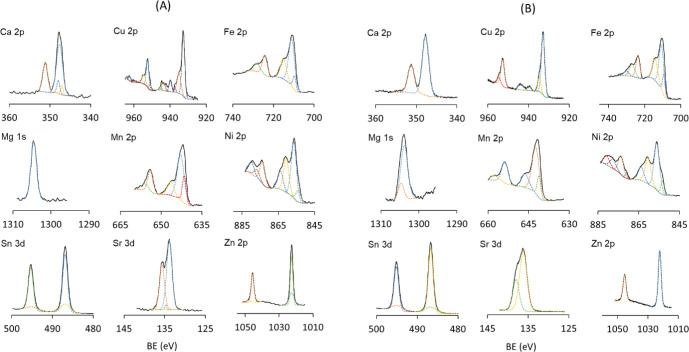
High-resolution spectra of PSPMA (A) and PAA (B) brushes after incubation in 10 mM aqueous solutions containing various chloride salts with varying cations. Dashed colored lines represent individual fitted components corresponding to distinct chemical environments; the black solid line corresponds to the data.

For both brush types, the high-resolution spectra of each cation, except for Mg, exhibit spin–orbit splitting (or j–j coupling). This phenomenon is usually observed for 2p spectra, which present two spin–orbit peaks (2*p*_1/2_ and 2p_3/2_) with a typical area ratio of 1:2 (corresponding to 2 electrons in the 2p_1/2_ level and 4 electrons in the 2p_3/2_ level).^83^ The doublets are also present in the Sr spectrum; however, they are overlapping. The high-resolution spectra of copper, iron, manganese, and nickel show strong satellite features next to the main peaks, which are usually associated with shakeups, energy loss, plasmons, and other unidentified peaks. These asymmetric peak shapes, with a tail at higher binding energies and shakeup features, result from a de-excitation process, where the outgoing core electrons interact with valence electrons and excite them to a higher energy level.^83^ The peak fitting is much more complicated for these spectra due to the presence of hidden satellites and larger background; however, these features are usually observed for species with II and III valence.^84^

The spectra of Sn 3d, Zn 2p, and Ca 2p present symmetric peaks and do not display any satellite features, although looking at their Auger parameters could help identify possible hidden signals. The wide scans of the samples (Figures S1–S18) also display several strong peaks associated with the electron configuration of each cation adsorbed onto the surface. From the atomic percentages in the tables of the wide scans of PSPMA samples (Table 1), the ratio between divalent cations and sulfur from the SO_3_^–^ groups was calculated to be always ∼1:2 or higher (except for tin). This suggests that each adsorbed ion likely interacts with at least two SO_3_^–^ groups concurrently, supporting earlier hypotheses of interchain cross-linking or reduced chain mobility when in solution.

**Table 1:** Atomic % of Sulfur in PSPMA Brushes and the Cations from the Different Salt Solutions (after Incubation), Measured by XPS

Cation in solution	Atomic % of the cation (2p1) in the PSPMA brush after incubation	Atomic % of S (2p) of the PSPMA brush after incubation in the respective cation solution	Ratio of the atomic % of S:cation
Sn^2+^	10.12^a^	1.48	0.15
Mn^2+^	3.30	7.30	2.21
Zn^2+^	3.36	6.67	1.99
Ca^2+^	2.87	6.31	2.20
Cu^2+^	2.30	6.46	2.81
Ni^2+^	2.15	5.91	2.75
Fe^2+^	2.85	6.23	2.19
Sr^2+^	2.82^a^	7.34	2.60
Mg^2+^	2.87	7.05	2.46

a For Sn^2+^ and Sr^2+^, the value of atomic % was taken by their 3d signal, as suggested by the XPS software.

Considering the distinct swelling and collapse behaviors observed in PSPMA and PAA brushes (linked to differences in hydration energies), we sought to explore whether the counter-anions of the electrolyte introduced in corresponding aqueous solutions might also influence brush behavior. Although anionic brushes are generally studied with a focus on cation effects, we hypothesized that anions could play a more active role than commonly assumed (due to the assumption that like-charged species would exert negligible effects). In situ ellipsometry was therefore used to evaluate the impact of different anions on PSPMA and PAA brush conformation (Figure 7), using a panel of sodium salts with corresponding counterions spanning the Hofmeister series (ClO_4_^–^, NO_3_^–^, Cl^–^, F^–^), across a range of ionic strengths.

**Figure 7:**
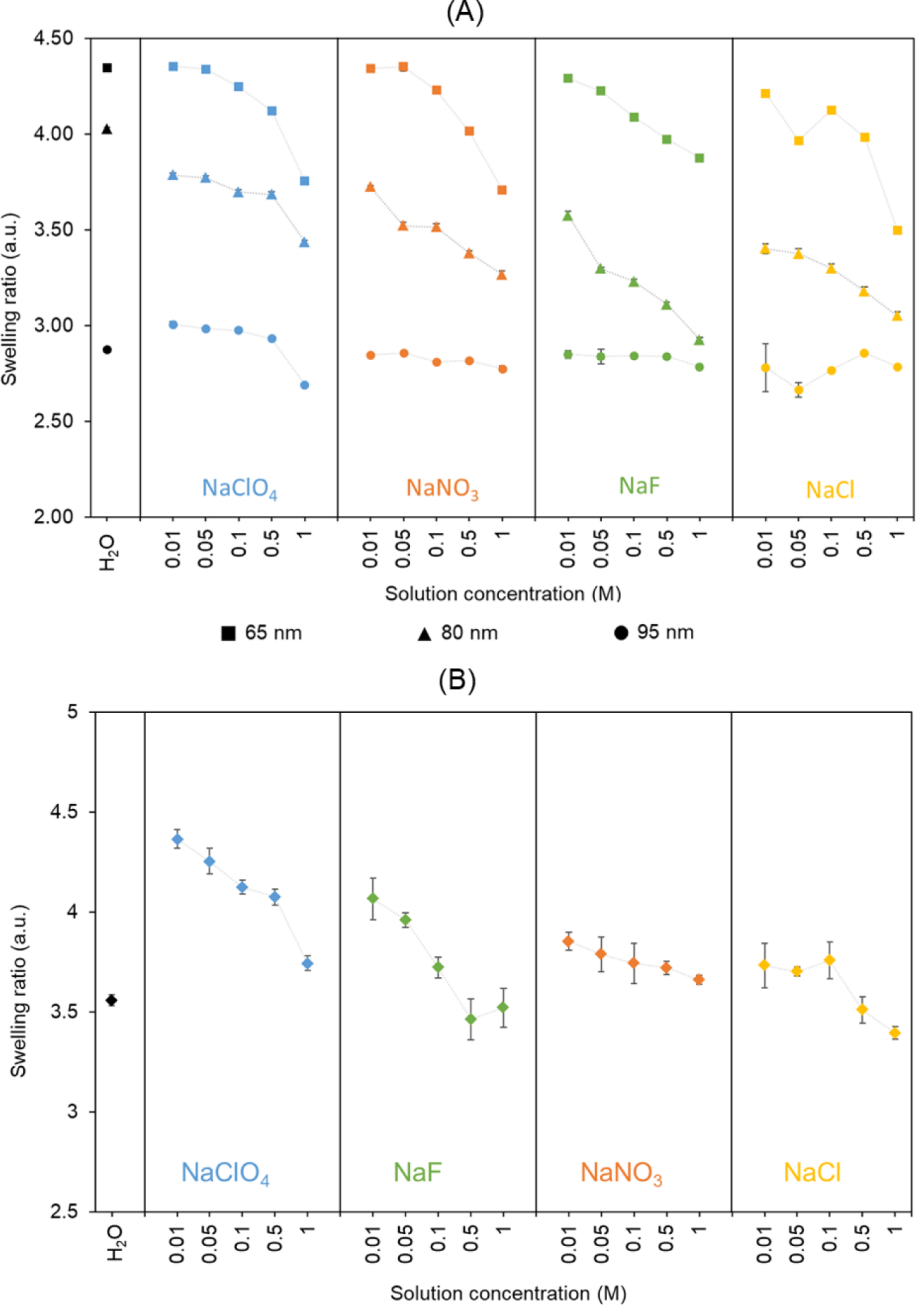
Swelling ratios of (A) PSPMA brushes (with three different initial thicknesses) and (B) PAA brushes, calculated from in situ ellipsometry measurements conducted in deionized water, NaClO_4_, NaNO_3_, NaF, and NaCl solutions with varying salt concentrations (0.01 to 1 M).

Both brushes collapsed gradually as a function of increasing electrolyte concentration, reflecting progressively higher electrostatic shielding and an associated salting-out effect.^85^ For PSPMA, the swelling of the brushes was slightly higher in deionized water than in salt-containing solutions, even at low ionic strength (10 mM). This behavior is expected, as PSPMA possesses permanently ionized sulfonate groups with counterions already present within the brush and partially in the surrounding diffuse layer, as discussed by Ballauff and Borisov.^20^ The small difference in swelling between deionized water and low salt conditions can also be attributed to the exchange of the original potassium counterions with sodium ions during EDTA treatment, resulting in no net change in the counterion environment upon the addition of sodium salts. It was also observed that PSPMA brushes exhibited greater swelling in the presence of monovalent cations such as Na^+^ compared to divalent cations, in agreement with Kou et al.^42^ This increase in swelling can be attributed to the inability of Na^+^ (being monovalent) to electrostatically cross-link polymer segments, which as a result are less constrained and can more easily extend. Hence, the effect of sodium ions on brush swelling was considered to result solely from charge screening. Nonetheless, the type of anion associated with Na^+^ showed a measurable influence on PSPMA brush behavior. Solutions containing ClO_4_^–^, a chaotropic anion, induced higher swelling than those with kosmotropic anions such as Cl^–^ and F^–^, following the order ClO_4_^–^ > NO_3_^–^ > Cl^–^ ∼ F^–^. This was likely due to differences in hydration: weakly hydrated anions such as ClO_4_^–^ increased the availability of water to the brushes, indirectly enhancing their swelling.

In PAA brushes, however, swelling was observed to be higher in low-salt solutions compared to deionized water, a behavior attributed to cation–proton exchange at the carboxylic acid groups, which increases brush ionization and internal osmotic pressure. This effect was particularly evident in the NaClO_4_ series and weakest with NaCl, with a significant jump in swelling at low ionic strength, compared to deionized water, prior to a gradual collapse at higher concentrations. Therefore, the anion identity also influenced PAA swelling, broadly following the trend ClO_4_^–^ > F^–^ > NO_3_^–^ > Cl^–^, although the differences between F^–^, NO_3_^–^, and Cl^–^ were less distinct, likely due to their similar hydration characteristics.

## Conclusions

PAA and PSPMA polymer brushes were synthesized on silicon substrates and characterized via ellipsometry, in situ ellipsometry, and XPS to investigate their structural and swelling behaviors under varying conditions. The impact of residual copper catalysts on swelling and ionic interactions was analyzed for both brushes, and a 10 mM EDTA treatment was applied to effectively remove these residues. For PSPMA brushes, the thickness was systematically varied via controlled polymerization to assess its influence on swelling. In contrast, PAA brushes were evaluated at a single fixed thickness.

In aqueous chloride salt solutions containing divalent cations, PSPMA brushes (being strong polyelectrolytes) exhibited ion-specific collapse. Cations characterized by lower absolute hydration enthalpies, larger ionic radii, and a more chaotropic character (Sn^2+^, Zn^2+^, Sr^2+^, Ca^2+^, Mn^2+^) induced greater deswelling, while those with higher absolute hydration enthalpies, smaller radii, and a more kosmotropic character (Ni^2+^, Mg^2+^, Cu^2+^, Fe^2+^) caused a less pronounced collapse. Deviations for Zn^2+^ and Sn^2+^ were attributed to the formation of insoluble species within the brush layer. PAA brushes, in contrast, exhibited a more pronounced pH-sensitive response, characteristic of weak polyelectrolytes. While some distinctions between chaotropic (Sn^2+^, Zn^2+^, Mn^2+^) and kosmotropic (Fe^2+^, Sr^2+^, Mg^2+^) cations were observed, the swelling behavior of PAA often deviated from trends predicted by the Hofmeister series or by correlations with hydration enthalpy and ionic radius, highlighting the dominant influence of pH-driven ionization.

The effect of different anionic species on the brushes’ swelling behavior was also evaluated using a series of sodium salts. Although their influence was less pronounced than that of divalent cations, the swelling responses of both PAA and PSPMA brushes varied depending on the kosmotropic or chaotropic character of the sodium counterions. This was attributed to changes in the water structure and availability, which in turn modulated the hydration environment of the brushes and indirectly influenced their swelling.

Lastly, XPS confirmed that both PAA and PSPMA brushes were capable of significantly absorbing and retaining all nine cationic species studied, strengthening their relevance for applications involving ion uptake, selective binding, and surface postfunctionalization.

## Supplementary Material



## Notes

Details of PSPMA brush growth kinetics, optical constants and models, XPS spectra of PAA and PSPMA brushes, and additional swelling behavior data; statistical analyses, along with ion-specific studies of cation and anion series for both PAA and PSPMA brushes (PDF)

## References

[1] Robertson H., Johnson E. C., Gresham I. J., Prescott S. W., Nelson A., Wanless E. J., Webber G. B. (2021). Competitive Specific Ion Effects
in Mixed Salt Solutions on a Thermoresponsive Polymer Brush. J. Colloid Interface Sci..

[2] Gregory K. P., Elliott G. R., Robertson H., Kumar A., Wanless E. J., Webber G. B., Craig V. S. J., Andersson G. G., Page A. J. (2022). Understanding Specific Ion Effects
and the Hofmeister
Series. Phys. Chem. Chem. Phys..

[3] Zimmermann R., Gunkel-Grabole G., Bünsow J., Werner C., Huck W. T. S., Duval J. F. L. (2017). Evidence
of Ion-Pairing in Cationic Brushes from Evaluation
of Brush Charging and Structure by Electrokinetic and Surface Conductivity
Analysis. J. Phys. Chem. C.

[4] Dahman, Y. K. A. B. D. Smart Nanomaterials. In Nanotechnology and Functional Materials for Engineers; Elsevier, 2017; pp. 47–66.

[5] Willott J. D., Murdoch T. J., Webber G. B., Wanless E. J. (2017). Physicochemical
Behaviour of Cationic Polyelectrolyte Brushes. Prog. Polym. Sci..

[6] Krishnamoorthy M., Hakobyan S., Ramstedt M., Gautrot J. E. (2014). Surface-Initiated
Polymer Brushes in the Biomedical Field: Applications in Membrane
Science, Biosensing, Cell Culture, Regenerative Medicine and Antibacterial
Coatings. Chem. Rev..

[7] Li D., Sharili A. S., Connelly J., Gautrot J. E. (2018). Highly Stable RNA
Capture by Dense Cationic Polymer Brushes for the Design of Cytocompatible,
Serum-Stable SiRNA Delivery Vectors. Biomacromolecules.

[8] Raynold A. A. M., Li D., Chang L., Gautrot J. E. (2021). Competitive Binding
and Molecular Crowding Regulate the Cytoplasmic Interactome of Non-Viral
Polymeric Gene Delivery Vectors. Nat. Commun..

[9] Guo S., Jańczewski D., Zhu X., Quintana R., He T., Neoh K. G. (2015). Surface Charge Control
for Zwitterionic Polymer Brushes:
Tailoring Surface Properties to Antifouling Applications. J. Colloid Interface Sci..

[10] Chen T., Ferris R., Zhang J., Ducker R., Zauscher S. (2010). Stimulus-Responsive
Polymer Brushes on Surfaces: Transduction Mechanisms and Applications. Prog. Polym. Sci..

[11] Bünsow J., Kelby T. S., Huck W. T. S. (2010). Polymer
Brushes: Routes toward Mechanosensitive
Surfaces. Acc. Chem. Res..

[12] Metze F. K., Sant S., Meng Z., Klok H.-A., Kaur K. (2023). Swelling-Activated
Soft Mechanochemistry in Polymer Materials. Langmuir.

[13] Qu F., Li D., Ma X., Chen F., Gautrot J. E. (2019). A Kinetic
Model
of Oligonucleotide-Brush Interactions for the Rational Design of Gene
Delivery Vectors. Biomacromolecules.

[14] Li D., Xu L., Wang J., Gautrot J. E. (2021). Responsive Polymer Brush Design and
Emerging Applications for Nanotheranostics. Adv. Healthcare Mater..

[15] Das S., Banik M., Chen G., Sinha S., Mukherjee R. (2015). Polyelectrolyte
Brushes: Theory, Modelling, Synthesis and Applications. Soft Matter..

[16] Toomey R., Tirrell M. (2008). Functional Polymer
Brushes in Aqueous Media from Self-Assembled
and Surface-Initiated Polymers. Annu. Rev. Phys.
Chem..

[17] Spain, S. G. ; Yaşayan, G. ; Soliman, M. ; Heath, F. ; Saeed, A. O. ; Alexander, C. Nanoparticles for Nucleic Acid Delivery. In Comprehensive Biomaterials, Ducheyne, Paul , Eds.; Elsevier: Oxford, 2011; pp. 389–410.

[18] Franck-Lacaze L., Sistat P., Huguet P. (2009). Determination of the
PK­(a) of Poly
(4-Vinylpyridine)-Based Weak Anion Exchange Membranes for the Investigation
of the Side Proton Leakage. J. Membr. Sci..

[19] Matsen M. W. (2011). Compression
of Polyelectrolyte Brushes in a Salt-Free Theta Solvent. Eur. Phys. J. E.

[20] Ballauff M., Borisov O. (2006). Polyelectrolyte Brushes. Curr.
Opin. Colloid Interface Sci..

[21] Ehtiati K., Moghaddam S. Z., Klok H. A., Daugaard A. E., Thormann E. (2022). Specific Counterion
Effects on the Swelling Behavior of Strong Polyelectrolyte Brushes. Macromolecules.

[22] Israels R., Leermakers F. A. M., Fleer G. J. (1994). On the Theory of Grafted Weak Polyacids. Macromolecules.

[23] Kunz W., Henle J., Ninham B. W. (2004). ‘Zur Lehre von Der Wirkung
Der Salze’ (about the Science of the Effect of Salts): Franz
Hofmeister’s Historical Papers. Curr.
Opin. Colloid Interface Sci..

[24] Zangi R. (2010). Can Salting-In/Salting-Out
Ions Be Classified as Chaotropes/Kosmotropes?. J. Phys. Chem. B.

[25] Lo
Nostro P., Ninham B. W. (2012). Hofmeister Phenomena: An Update on
Ion Specificity in Biology. Chem. Rev..

[26] Record M.
T., Guinn E., Pegram L., Capp M. (2013). Introductory Lecture:
Interpreting and Predicting Hofmeister Salt Ion and Solute Effects
on Biopolymer and Model Processes Using the Solute Partitioning Model. Faraday Discuss.

[27] Mazzini V., Craig V. S. J. (2017). What Is the Fundamental Ion-Specific Series for Anions
and Cations? Ion Specificity in Standard Partial Molar Volumes of
Electrolytes and Electrostriction in Water and Non-Aqueous Solvents. Chem. Sci..

[28] Tan K. Y., Gautrot J. E., Huck W. T. S. (2011). Formation
of Pickering Emulsions
Using Ion-Specific Responsive Colloids. Langmuir.

[29] Willott J.
D., Murdoch T. J., Humphreys B. A., Edmondson S., Wanless E. J., Webber G. B. (2015). Anion-Specific
Effects on the Behavior
of PH-Sensitive Polybasic Brushes. Langmuir.

[30] Wei Q., Cai M., Zhou F., Liu W. (2013). Dramatically Tuning Friction Using
Responsive Polyelectrolyte Brushes. Macromolecules.

[31] Hegaard F., Biro R., Ehtiati K., Thormann E. (2023). Ion-Specific Antipolyelectrolyte
Effect on the Swelling Behavior of Polyzwitterionic Layers. Langmuir.

[32] Murdoch T. J., Humphreys B. A., Johnson E. C., Prescott S. W., Nelson A., Wanless E. J., Webber G. B. (2018). The Role of Copolymer Composition
on the Specific Ion and Thermo-Response of Ethylene Glycol-Based Brushes. Polymer.

[33] Murdoch T. J., Humphreys B. A., Johnson E. C., Webber G. B., Wanless E. J. (2018). Specific
Ion Effects on Thermoresponsive Polymer Brushes: Comparison to Other
Architectures. J. Colloid Interface Sci..

[34] Alonso-García T., Gervasi C. A., Rodríguez-Presa M. J., Gutiérrez-Pineda E., Moya S. E., Azzaroni O. (2013). Temperature-Dependent Transport Properties
of Poly­[2-(Methacryloyloxy) Ethyl]­Trimethylammonium Chloride Brushes
Resulting from Ion Specific Effects. J. Phys.
Chem. C.

[35] Flemming P., Müller M., Fery A., Münch A. S., Uhlmann P. (2020). Mechanistic Investigation of the Counterion-Induced
UCST Behavior of Poly­(N, N-Dimethylaminoethyl Methacrylate) Polymer
Brushes. Macromolecules.

[36] Rodríguez-Ropero F., van der
Vegt N. F. A. (2013). Ionic Specific Effects on the Structure, Mechanics
and Interfacial Softness of a Polyelectrolyte Brush. Faraday Discuss..

[37] Wang T., Long Y., Liu L., Wang X., Craig V. S. J., Zhang G., Liu G. (2014). Cation-Specific Conformational Behavior
of Polyelectrolyte Brushes: From Aqueous to Nonaqueous Solvent. Langmuir.

[38] Rzhepishevska O., Hakobyan S., Ruhal R., Gautrot J., Barbero D., Ramstedt M. (2013). The Surface Charge
of Anti-Bacterial Coatings Alters
Motility and Biofilm Architecture. Biomater.
Sci..

[39] Li P., Ding Z., Yin Y., Yu X., Yuan Y., Brió Pérez M., de Beer S., Vancso G. J., Yu Y., Zhang S. (2020). Cu2 ± Doping of Polyanionic Brushes: A Facile
Route to Prepare Implant Coatings with Both Antifouling and Antibacterial
Properties. Eur. Polym. J..

[40] Marchena M. H., Lambert E., Bogdanović B., Quadir F., Neri-Cruz C. E., Luo J., Nadal C., Migliorini E., Gautrot J. E. (2024). BMP-Binding Polysulfonate
Brushes to Control Growth Factor Presentation and Regulate Matrix
Remodelling. ACS Appl. Mater. Interfaces.

[41] He Z., Xie W. J., Liu Z., Liu G., Wang Z., Gao Y. Q., Wang J. (2016). Tuning Ice
Nucleation with Counterions
on Polyelectrolyte Brush Surfaces. Sci. Adv..

[42] Kou R., Zhang J., Wang T., Liu G. (2015). Interactions between
Polyelectrolyte Brushes and Hofmeister Ions: Chaotropes versus Kosmotropes. Langmuir.

[43] Wang T., Kou R., Zhang J., Zhu R., Cai H., Liu G. (2020). Tuning the
Light Response of Strong Polyelectrolyte Brushes with Counterions. Langmuir.

[44] Xu C., Yan Y., Tan J., Yang D., Jia X., Wang L., Xu Y., Cao S., Sun S. (2019). Biodegradable Nanoparticles of Polyacrylic
Acid–Stabilized Amorphous CaCO3 for Tunable PH-Responsive Drug
Delivery and Enhanced Tumor Inhibition. Adv.
Funct. Mater..

[45] Chen Q., Wang J., Chen K., Zhang R., Li L., Guo X. (2014). Heavy Metal Ions Removal by Nano-Sized Spherical Polymer Brushes. Chin. J. Polym. Sci..

[46] Dagand L., Urban B., Li Q., Hermes I. M., Bittrich E., Uhlmann P., Müller M., Münch A. S. (2025). Interaction
of Poly­(Acrylic Acid) Brushes with Multivalent Cations: An In Situ
Study. Langmuir.

[47] Hollmann O., Czeslik C. (2006). Characterization of
a Planar Poly­(Acrylic Acid) Brush
as a Materials Coating for Controlled Protein Immobilization. Langmuir.

[48] Mahendran V., Philip J. (2013). Sensing of Biologically
Important Cations Such as Na+,
K+, Ca2+, Cu2+, and Fe3+ Using Magnetic Nanoemulsions. Langmuir.

[49] Kiriukhin M., Collins K. (2002). Dynamic Hydration Numbers
for Biologically Important
Ions. Biophys. Chem..

[50] Addy M., Greenman J., Renton-Harper P., Newcombe R., Doherry F. (1997). Studies on
Stannous Fluoride Toothpaste and Gel (2). Effects on Salivary Bacterial
Counts and Plaque Regrowth in Vivo. J. Clin.
Periodontol..

[51] Cozens E. J., Kong D., Roohpour N., Gautrot J. E. (2020). The Physico-Chemistry
of Adhesions of Protein Resistant and Weak Polyelectrolyte Brushes
to Cells and Tissues. Soft Matter..

[52] Lego B., François M., Skene W. G., Giasson S. (2009). Polymer Brush Covalently
Attached to OH-Functionalized Mica Surface via Surface-Initiated ATRP:
Control of Grafting Density and Polymer Chain Length. Langmuir.

[53] Wu T., Gong P., Szleifer I., Vlček P., Šubr V., Genzer J. (2007). Behavior of Surface-Anchored
Poly­(Acrylic
Acid) Brushes with Grafting Density Gradients on Solid Substrates:
1. Experiment. Macromolecules.

[54] Guo S., Quintana R., Cirelli M., Toa Z. S. D., Arjunan
Vasantha V., Kooij E. S., Jańczewski D., Vancso G. J. (2019). Brush Swelling and Attachment Strength of Barnacle
Adhesion Protein on Zwitterionic Polymer Films as a Function of Macromolecular
Structure. Langmuir.

[55] Malham I. B., Bureau L. (2010). Density Effects on
Collapse, Compression, and Adhesion
of Thermoresponsive Polymer Brushes. Langmuir.

[56] Jia H., Wildes A., Titmuss S. (2012). Structure
of PH-Responsive Polymer
Brushes Grown at the Gold-Water Interface: Dependence on Grafting
Density and Temperature. Macromolecules.

[57] Plunkett K.
N., Zhu X., Moore J. S., Leckband D. E. (2006). PNIPAM Chain Collapse Depends on
the Molecular Weight and Grafting Density. Langmuir.

[58] Chu X., Yang J., Liu G., Zhao J. (2014). Swelling Enhancement
of Polyelectrolyte Brushes Induced by External Ions. Soft Matter.

[59] Brittain W. J., Minko S. (2007). A Structural Definition
of Polymer Brushes. J. Polym. Sci., Part A:
polym. Chem..

[60] Laurent P., Souharce G., Duchet-Rumeau J., Portinha D., Charlot A. (2012). “Pancake”
vs. Brush-like Regime of Quaternizable Polymer Grafts: An Efficient
Tool for Nano-Templating Polyelectrolyte Self-Assembly. Soft Matter..

[61] Ramstedt M., Cheng N., Azzaroni O., Mossialos D., Mathieu H. J., Huck W. T. S. (2007). Synthesis and Characterization of
Poly­(3-Sulfopropylmethacrylate) Brushes for Potential Antibacterial
Applications. Langmuir.

[62] Louette P., Bodino F., Pireaux J.-J. (2005). Poly­(Acrylic
Acid) (PAA) XPS Reference
Core Level and Energy Loss Spectra. Surf. Sci.
Spectra.

[63] Chaurin V., Constable E. C., Housecroft C. E. (2006). What Is the Coordination Number of
Copper­(Ii) in Metallosupramolecular Chemistry?. New J. Chem..

[64] Wilkinson, G. ; Gillard, R. D. ; McCleverty, J. A. Comprehensive Coordination Chemistry: The Synthesis, Reactions, Properties and Applications of Coordination Compounds.; Pergamon Press, 1987, Vol. 3, pp. 1–1601.

[65] Miura S., Shidara Y., Yunoki T., Mamun M. A. A., Shibasaki Y., Fujimori A. (2017). High-Density Packing of Amorphous Polymer with Bulky
Aromatic Rings in Interfacial Molecular Films. Macromol. Chem. Phys..

[66] Romio M., Grob B., Trachsel L., Mattarei A., Morgese G., Ramakrishna S. N., Niccolai F., Guazzelli E., Paradisi C., Martinelli E., Spencer N. D., Benetti E. M. (2021). Dispersity
within Brushes Plays a Major Role in Determining Their Interfacial
Properties: The Case of Oligoxazoline-Based Graft Polymers. J. Am. Chem. Soc..

[67] Yu Y., Cirelli M., Li P., Ding Z., Yin Y., Yuan Y., De Beer S., Vancso G. J., Zhang S. (2019). Enhanced Stability
of Poly­(3-Sulfopropyl Methacrylate Potassium) Brushes Coated on Artificial
Implants in Combatting Bacterial Infections. Ind. Eng. Chem. Res..

[68] Sant S., Kaur K., Klok H. A. (2024). Swelling
and Degrafting of Poly­(3-Sulfopropyl
Methacrylate) Brushes. Langmuir.

[69] Li Y., Lin Y., Dai Y., Ko Y., Genzer J. (2019). Mechanochemical Degrafting
of a Surface-Tethered Poly­(Acrylic Acid) Brush Promoted Etching of
Its Underlying Silicon Substrate. Langmuir.

[70] Sudre G., Siband E., Hourdet D., Creton C., Cousin F., Tran Y. (2012). Synthesis and Characterization of Poly­(Acrylic Acid) Brushes: “Grafting-Onto”
Route. Chem.Phys..

[71] Karim A., Douglas J. F., Horkay F., Fetters L. J., Satija S. K. (1996). Comparative
Swelling of Gels and Polymer Brush Layers. Phys.
B.

[72] Gong K., Marshall B. D., Chapman W. G. (2013). Modeling
Lower Critical Solution
Temperature Behavior of Associating Polymer Brushes with Classical
Density Functional Theory. J. Chem. Phys..

[73] Guptha V.
S., Hsiao P. Y. (2014). Polyelectrolyte
Brushes in Monovalent and Multivalent
Salt Solutions. Polymer.

[74] Orski S. V., Sheridan R. J., Chan E. P., Beers K. L. (2015). Utilizing Vapor
Swelling of Surface-Initiated Polymer Brushes to Develop Quantitative
Measurements of Brush Thermodynamics and Grafting Density. Polymer.

[75] Yadav V., Jaimes-Lizcano Y. A., Dewangan N. K., Park N., Li T. H., Robertson M. L., Conrad J. C. (2017). Tuning Bacterial
Attachment and Detachment
via the Thickness and Dispersity of a PH-Responsive Polymer Brush. ACS Appl. Mater. Interfaces.

[76] Parsons D. F., Boström M., Nostro P. L., Ninham B. W. (2011). Hofmeister Effects:
Interplay of Hydration, Nonelectrostatic Potentials, and Ion Size. Phys. Chem. Chem. Phys..

[77] Arraez F. J., Van Steenberge P. H. M., D’hooge D. R. (2020). The Competition of Termination and
Shielding to Evaluate the Success of Surface-Initiated Reversible
Deactivation Radical Polymerization. Polymers.

[78] Smith D. W. (1977). Ionic Hydration
Enthalpies. J. Chem. Educ..

[79] Marcus Y. (1988). Ionic Radii
in Aqueous Solutions. Chem. Rev..

[80] Yu J., Mao J., Yuan G., Satija S., Jiang Z., Chen W., Tirrell M. (2016). Structure of Polyelectrolyte Brushes in the Presence
of Multivalent Counterions. Macromolecules.

[81] Peacock J. C., Peacock B. L. (1918). Deg Some Observations the Dissolving of Zinc Chloride
and Several Suggested Solvents. J. Am. Pharm.
Assoc..

[82] Randall M., Murakami S. (1930). The Free Energy of Stannous Hydroxyl Chloride and the
Activity Coefficient of Stannous Chloride and Stannous Ions. J. Am. Chem. Soc..

[83] Biesinger M. C., Payne B. P., Grosvenor A. P., Lau L. W. M., Gerson A. R., Smart R. S. C. (2011). Resolving Surface
Chemical States in XPS Analysis of
First Row Transition Metals, Oxides and Hydroxides: Cr, Mn, Fe, Co
and Ni. Appl. Surf. Sci..

[84] Moulder, J. F. ; Stickle, W. F. ; Sobol, W. M. ; Bomben, K. D. In Handbook of X-Ray Photoelectron Spectroscopy, Chastain, Jill Eds; Physical Electronics Division, Perkin-Elmer Corporation, 1992.

[85] Biesalski M., Johannsmann D., Rühe J. (2002). Synthesis and Swelling Behavior of
a Weak Polyacid Brush. J. Chem. Phys..

