# Supplementary material for: Anion- and Cation-Specific Response of the Aqueous Conformation of Strong and Weak Polyanionic Brushes
Source: Langmuir. 2025 Oct 28;41(44):29502–15. doi: 10.1021/acs.langmuir.5c03349 (PMC12613818; doi:10.1021/acs.langmuir.5c03349)
Supplement: Supplementary file 1 [file la5c03349_si_001.pdf]

Supporting Information

Anion and cation-specific response of the aqueous conformation of strong and weak polyanionic brushes

Authors:

Claudia Bertei[1], Emiliano Bilotti[2*], Julien E. Gautrot[1*]

Affiliations:

[1]School of Engineering and Materials Science, Queen Mary University of London, E1 4NS London, United Kingdom.

[2]Department of Aeronautics, Imperial College London, SW7 2AZ London, United Kingdom

[*]**Corresponding authors:** j.gautrot@qmul.ac.uk and e.bilotti@imperial.ac.uk

Number of pages: 22

Number of figures: 22

Number of tables: 8

Contents

S1. PSPMA brush growth kinetics 2

S2. Determination of optical constants and models of media for in situ ellipsometry studies 2

S3. XPS Spectra of PAA and PSPMA brushes in different salts 4

S3.1 PAA brushes 4

S3.2 PSPMA brushes 9

S4. Additional info on swelling behaviour of PSPMA and PAA brushes 14

S5. Statistical tests 16

S5.1 Cations series 16

S5.1.1 PAA brushes 16

S5.1.2 PSPMA brushes 17

S5.2 Anions series 19

S5.2.1 PAA brushes 19

S5.2.2 PSPMA brushes 19

S1. PSPMA brush growth kinetics

Table S1 - List of PSPMA brush thicknesses obtained from different solvent ratios and polymerisation times (PTs) using ellipsometry.

Volume ratio MeOH/dH$_2$O	Brush thickness at 20 min PT (nm)*	Brush thickness at 30 min PT (nm)*	Brush thickness at 60 min PT (nm)*
**2:1**	12.9	24.0	31.9
**1:1**	34.0	48.5	64.2
**1:2**	68.9	85.9	98.7
**1:3**	81.4	92.6	108.0
**1:4**	77.8	97. 6	113.1

*All measurements have a MSE < 5.

S2. Determination of optical constants and models of media for in situ ellipsometry studies

Various 10mM solutions containing chloride salts with different divalent cations were prepared to assess their impact on the swelling of PAA and PSPMA brushes. The cations chosen for the chloride salt series were: Mg^{2+}, Ca^{2+}, Mn^{2+}, Fe^{2+}, Ni^{2+}, Cu^{2+}, Zn^{2+}, Sr^{2+}, Sn^{2+}.

Solutions containing sodium salts with different anions were also prepared to assess their impact on the swelling of PAA and PSPMA brushes. A range of five different concentrations for each salt was prepared; the anions chosen for the sodium series were: F^-, Cl^-, NO_3^- and ClO_4^-.

Finally, a limited range of PEG400-based solutions was prepared subjected to the solubility of the compounds in the medium. All PEG-based solutions that contain salts required overnight stirring with a magnetic stirrer to dissolve completely and become transparent.

The optical parameters (A, B and C) of every solutions were acquired at a single wavelength of 632.8 nm (from a HeNe laser), at room temperature, using an ellipsometer equipped with an in-situ chamber. The refractive index n was calculated by the ellipsometer software using Cauchy's equation below:

$$n(\lambda) = A + \frac{B}{\lambda^2} + \frac{C}{\lambda^4}$$

With λ expressed in micrometers.

A list of all the solutions and their optical properties is shown in **Table S2**.

Table S2 – List of solutions prepared for swelling studies of PAA and PSPMA brushes and their optical characteristics measured via in-situ ellipsometry.

Material	Concentration	A	$B\ (\mu m^2)$	$C\ (\mu m^4)$	n
		**Cation series**			
$CaCl_2$	10mM	1.316	0.00409	-6.83E-05	1.326
$CuCl_2$	10mM	1.306	-0.00627	0.0012	1.298
$FeCl_2$	10mM	1.313	0.00336	2.49E-06	1.321
$MgCl_2$	10mM	1.320	0.00428	-0.0001	1.330
$MnCl_2$	10mM	1.318	0.00448	-0.00014	1.328
$NiCl_2$	10mM	1.319	0.00372	-3.96E-05	1.328
$SnCl_2$	10mM	1.317	0.00319	0.000105	1.326
$SrCl_2$	10mM	1.324	0.00436	-9.92E-05	1.334
$ZnCl_2$	10mM	1.313	0.00374	1.48E-05	1.322
H_2O	10mM	1.316	0.00248	1.98E-04	1.323
$CuCl_2$	10mM	1.306	-0.00627	0.0012	1.298
$CuCl_2$	50mM	1.317	0.00361	-9.83E-07	1.326
$CuCl_2$	100mM	1.327	0.00042	0.000416	1.331
$ZnCl_2$	1mM	1.322	0.00135	0.000274	1.327
$ZnCl_2$	5mM	1.323	0.00515	-0.00013	1.335
$ZnCl_2$	10mM	1.313	0.00374	1.48E-05	1.322
		**Anion series**			
NaCl	10mM	1.326	0.00458	-9.67E-05	1.337
	50mM	1.326	0.00355	7.74E-05	1.335
	100mM	1.328	0.00368	4.59E-05	1.337
	500mM	1.329	0.00323	0.000128	1.338
	1M	1.326	0.00349	9.55E-05	1.335
NaF	10mM	1.324	0.00277	0.000202	1.332
	50mM	1.326	0.00373	3.25E-05	1.336
	100mM	1.327	0.00435	-4.35E-05	1.338
	500mM	1.317	0.00211	0.000212	1.324
	1M	1.322	0.00175	0.00022219	1.322
$NaNO_3$	10mM	1.319	0.00361	1.20E-05	1.328
	50mM	1.317	0.0041	-2.22E-05	1.327
	100mM	1.314	0.00293	0.000123	1.322
	500mM	1.317	0.00410	-2.218E-05	1.317
	1M	1.324	0.00155	0.000269	1.324
$NaClO_4$	10mM	1.303	-0.00224	0.000704	1.302
	50mM	1.296	-0.00228	0.000791	1.295
	100mM	1.325	0.00234	0.000171	1.332
	500mM	1.329	0.00323	0.000128	1.338
	1M	1.332	0.00375	2.74E-05	1.342

S3. XPS Spectra of PAA and PSPMA brushes in different salts

List of wide scan and high resolution XPS spectra of PSPMA and PAA brushes incubated in various solutions containing different cations following the procedures described in the **Methods** section.

S3.1 PAA brushes

Table S3 – List of peaks from wide scan XPS spectra of PAA brushes before and after EDTA treatment.

Before EDTA			After EDTA		
Name	Peak BE	Atomic %	Name	Peak BE	Atomic %
C1s	286.09	66.18	C1s	284.8	58.78
O1s	533.38	31.05	O1s	531.99	35.83
N1s	402.81	2.77	Na1s	1071.46	2.87
			Na KL1	496.82	2.52

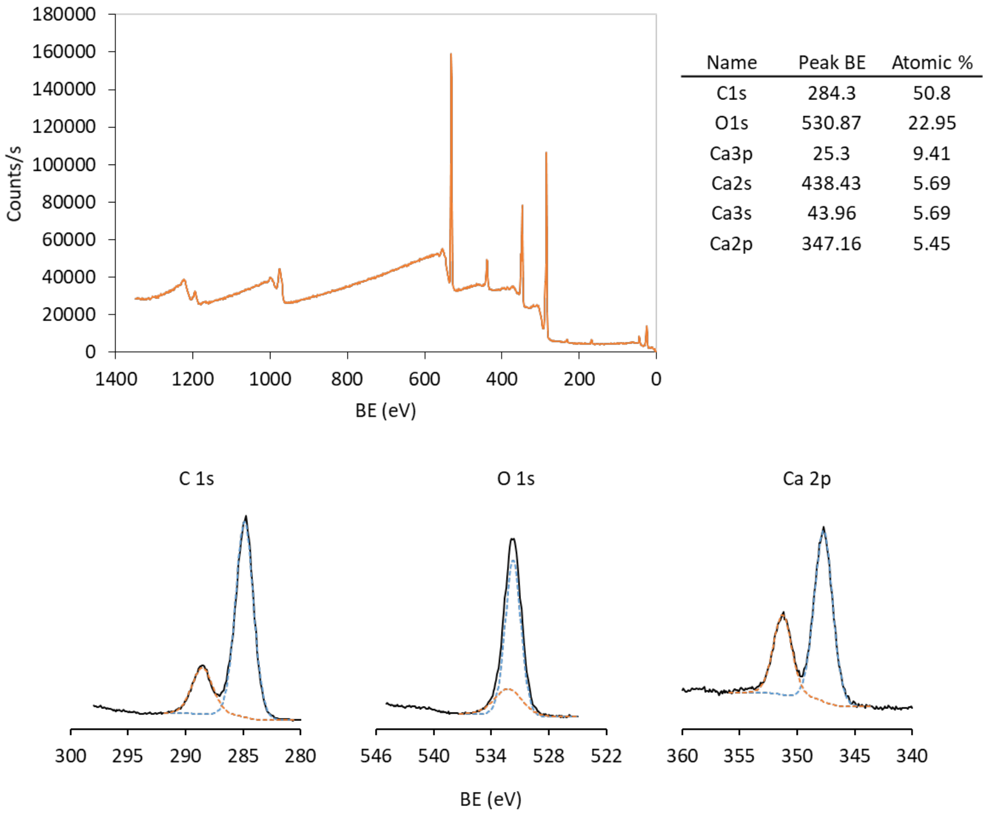

Figure S1 – Wide scan and high resolution XPS spectra of PAA brushes incubated in 10 mM $CaCl_2$

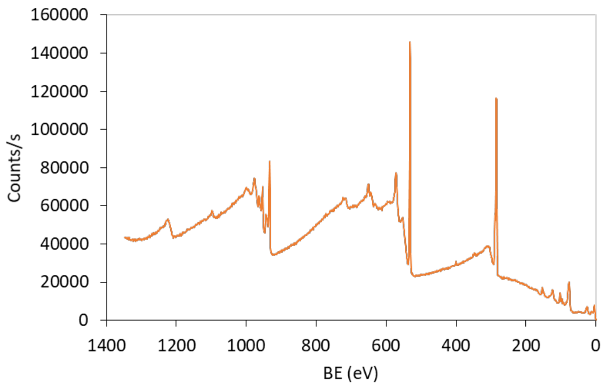

Name	Peak BE	Atomic %
C1s	284.5	49.07
O1s	531.82	22.58
Cu3p1	77.02	14.15
Cu3s	123.5	6.77
Cu2p3	933.43	3.68
Cu2p1	953.19	2.78
Cu2s	1097.8	0.96

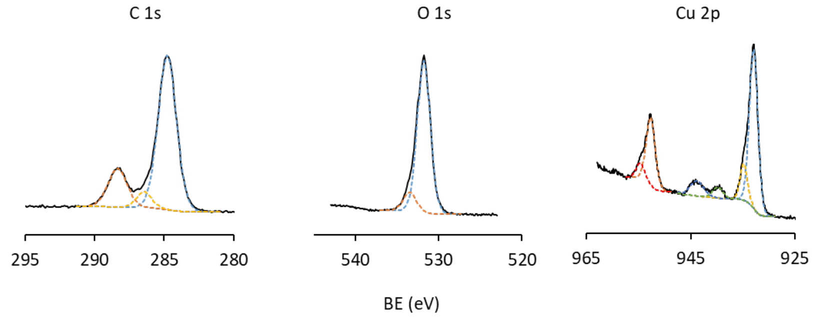

Figure S2 – Wide scan and high resolution XPS spectra of PAA brushes incubated in 10 mM $CuCl_2$

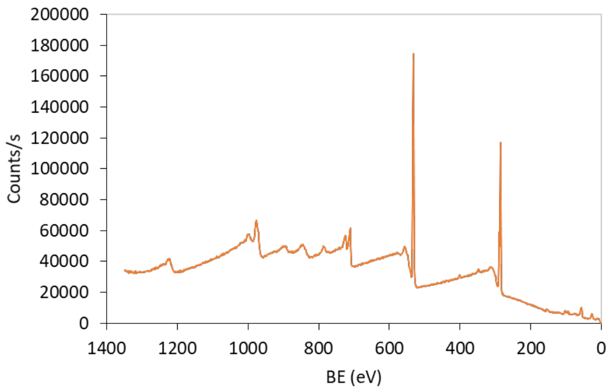

Name	Peak BE	Atomic %
C1s	285.16	57.75
O1s	532.18	33.72
Fe2p	711.28	3.95
Fe2s	847.04	3.01
Fe3p	55.79	1.57

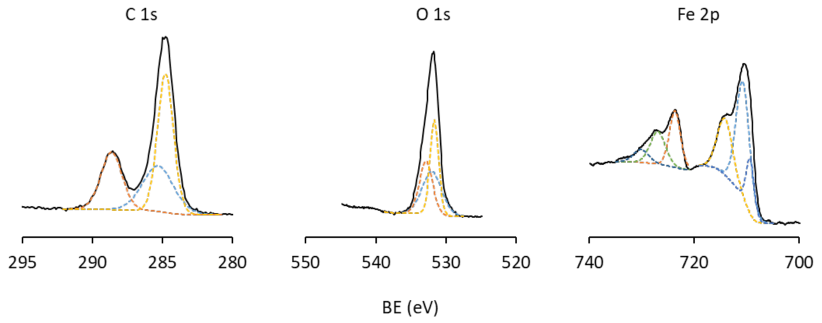

Figure S3 – Wide scan and high resolution XPS spectra of PAA brushes incubated in 10 mM $FeCl_2$ $4H_2O$

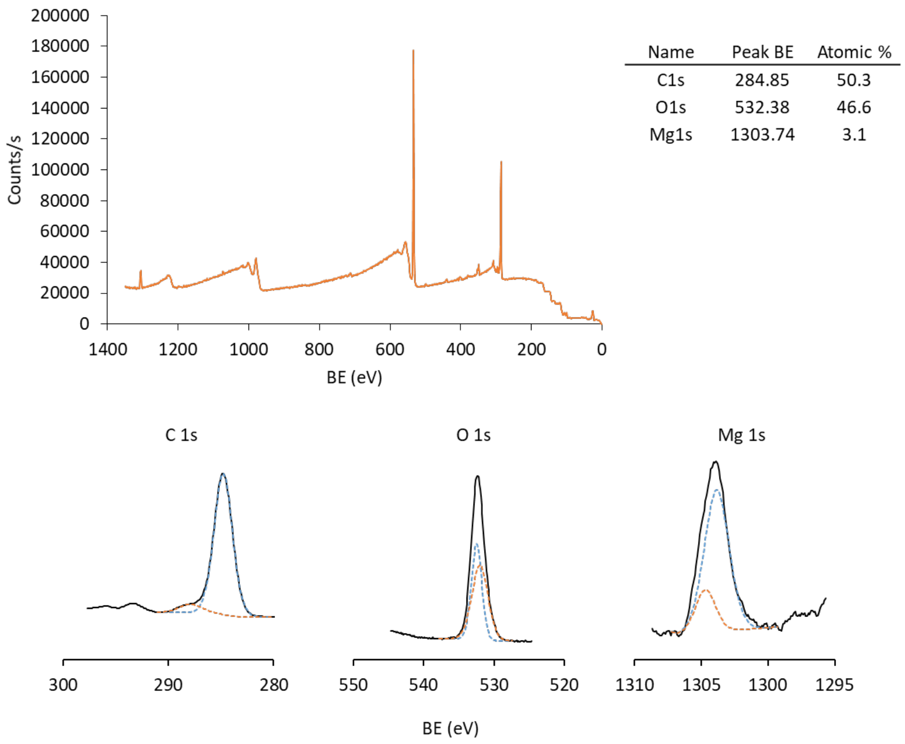

Figure S4 – Wide scan and high resolution XPS spectra of PAA brushes incubated in 10 mM $MgCl_2$

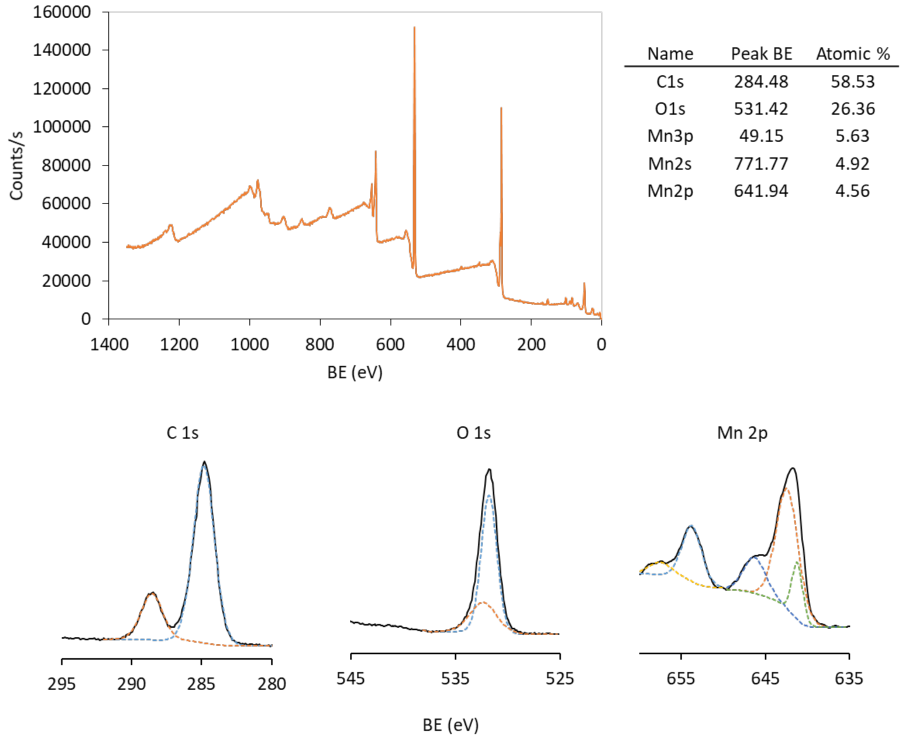

Figure S5 – Wide scan and high resolution XPS spectra of PAA brushes incubated in 10 mM $MnCl_2$ $4H_2O$

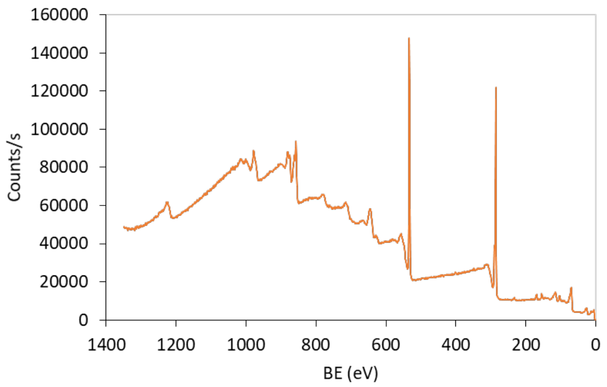

Name	Peak BE	Atomic %
C1s	285.03	61.34
O1s	531.96	27.34
Ni3p	69.44	4.95
Ni2p	856.28	4.59
Si2p	102.68	1.79

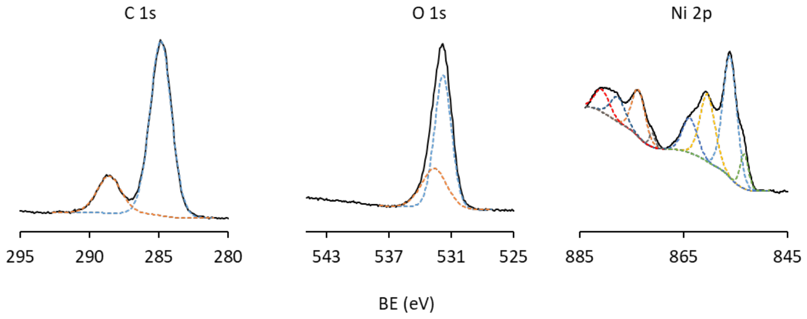

Figure S6 – Wide scan and high resolution XPS spectra of PAA brushes incubated in 10 mM $NiCl_2$

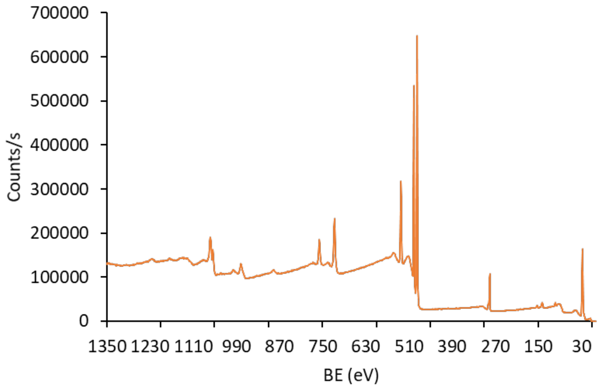

Name	Peak BE	Atomic %
C1s	284.4	29.26
O1s	531.03	27.75
Sn4d	26.08	10.29
Sn3d	486.36	9.58
Sn4s	138.63	8.65
Sn3p3	716.03	7.33
Sn3p1	757.98	7.15

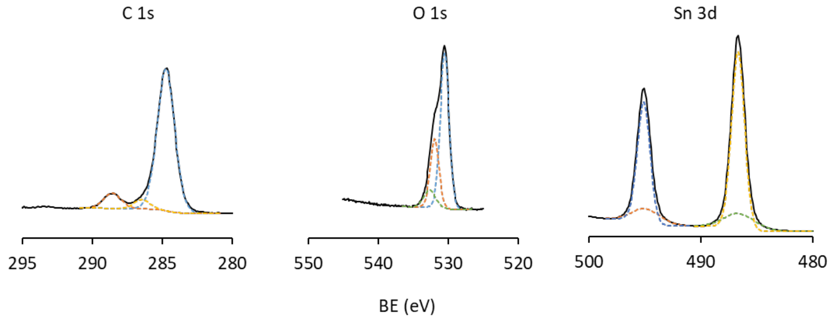

Figure S7 – Wide scan and high resolution XPS spectra of PAA brushes incubated in 10 mM $SnCl_2$

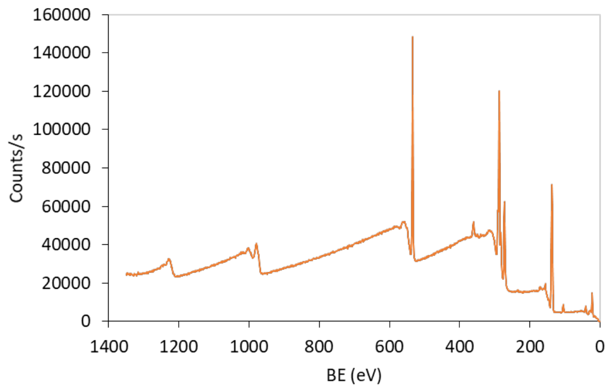

Name	Peak BE	Atomic %
C1s	284.3	54.73
O1s	531.06	20.18
Sr4s	37.87	8.32
Sr3s	357.73	4.41
Sr4p	19.42	4.28
Sr3d	134.13	4.12
Sr3p	269.06	2.29
Si2p	101.93	1.66

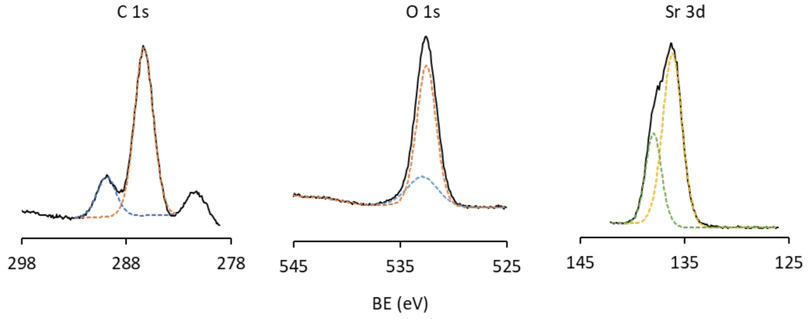

Figure S8 – Wide scan and high resolution XPS spectra of PAA brushes incubated in 10 mM $SrCl_2$ $6H_2O$

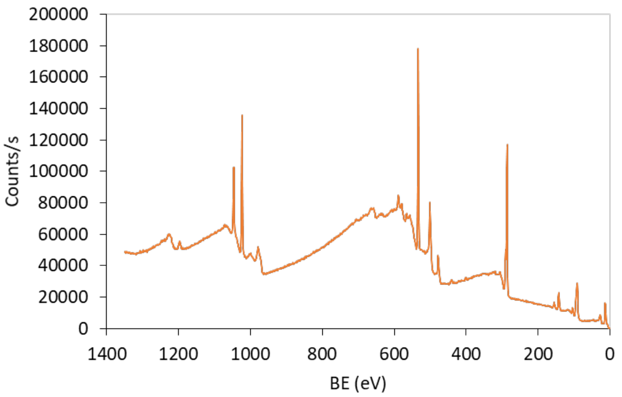

Name	Peak BE	Atomic %
C1s	284.8	35.5
O1s	531.92	26.31
Zn2p1	1022.03	17.05
Zn3p3	89.97	7.91
Zn3d	10.96	7.33
Zn3s	140.58	5.9

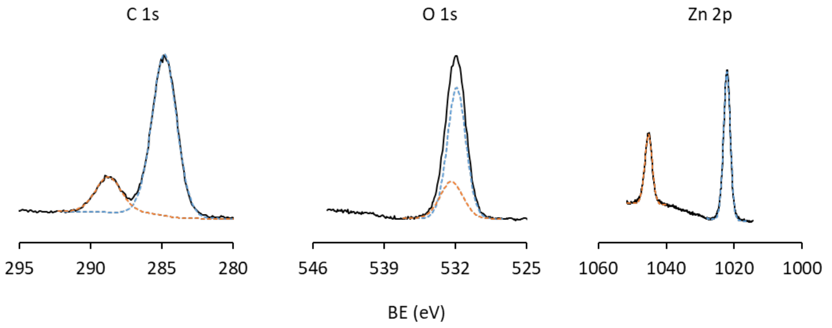

Figure S9 – Wide scan and high resolution XPS spectra of PAA brushes incubated in 10 mM $ZnCl_2$

S3.2 PSPMA brushes

Table S4 – List of peaks from wide scan XPS spectra of PSPMA brushes before and after EDTA treatment.

Before EDTA			After EDTA		
**Name**	**Peak BE**	**Atomic %**	**Name**	**Peak BE**	**Atomic %**
C1s	285.18	52.3	C1s	284.79	40.23
O1s	531.86	20.11	O1s	531.66	29.15
N1s	399.92	5.08	S2s	231.79	6.69
S2s	231.91	4.73	S2p	168.1	6.58
S2p	168.15	4.71	Na2s	63.01	6.15
Cu3p1	77.12	4.48	Na1s	1071.18	5.96
Cu LM6	719.3	3.63	Na KL1	497.07	5.24
Cu2p3	933.47	1.73			
Cu3s	123.24	1.32			
Cu2p1	953.08	1.26			
Cu LM3	651.49	0.64			

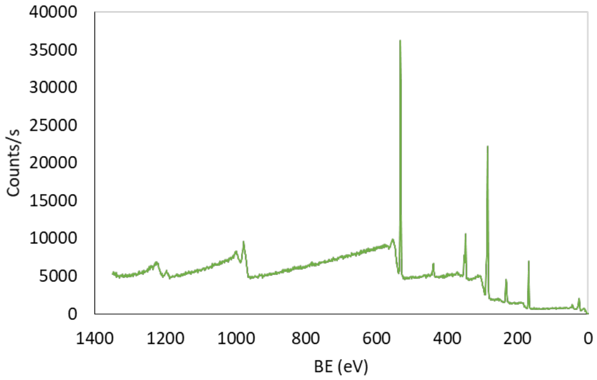

Name	Peak BE	Atomic %
C1s	285.04	44.58
O1s	532.01	27.05
Ca3p	25.33	6.56
S2p	168.31	6.31
S2s	232.05	6.28
Ca3s	44.09	3.42
Ca2s	439.24	2.93
Ca2p	347.97	2.87

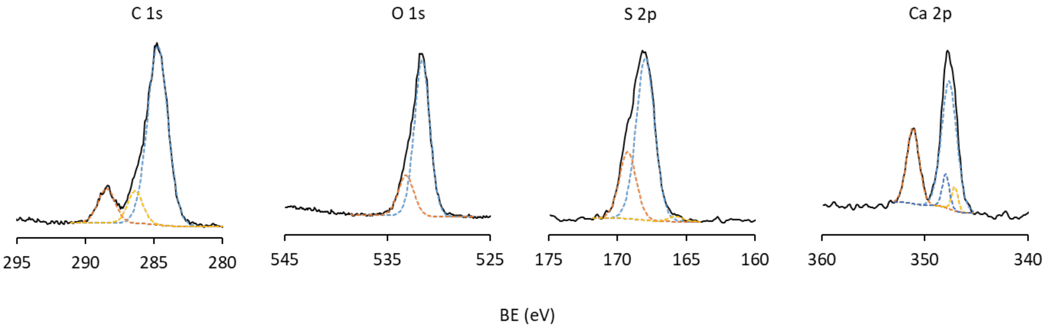

Figure S10 – Wide scan and high resolution XPS spectra of PAA brushes incubated in 10 mM $CaCl_2$

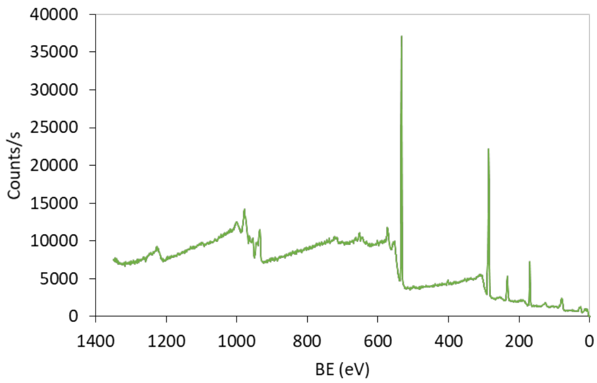

Name	Peak BE	Atomic %
C1s	286.05	47.28
O1s	533.08	28.9
S2p	169.41	6.87
S2s	233.18	6.46
Cu2p3	935.11	2.98
Cu3s	125.24	2.64
Cu3p	79.27	2.56
Cu2p1	954.29	2.3

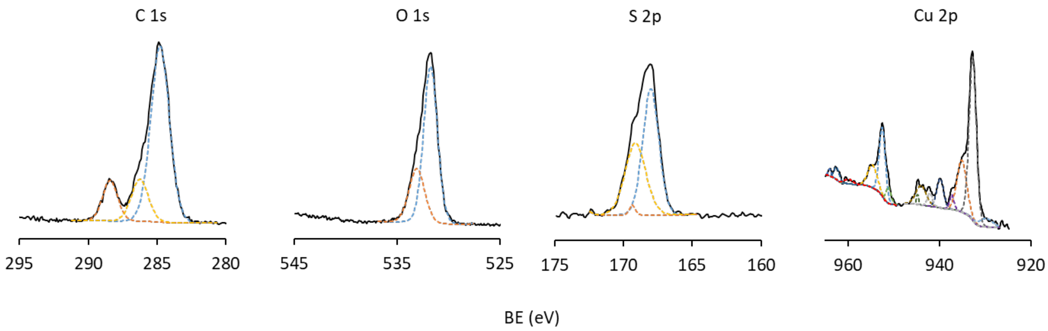

Figure S11 – Wide scan and high resolution XPS spectra of PAA brushes incubated in 10 mM $CuCl_2$

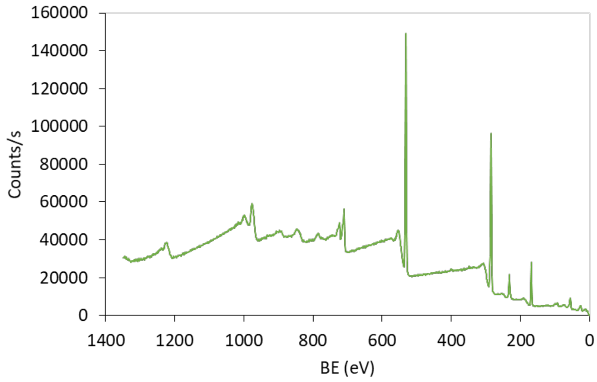

Name	Peak BE	Atomic %
C1s	284.9	49.48
O1s	531.35	26.99
S2p	168.44	6.23
Fe2s	844.12	6
S2s	232.12	5.99
Fe2p	710.28	2.85
Fe3p	55.92	2.46

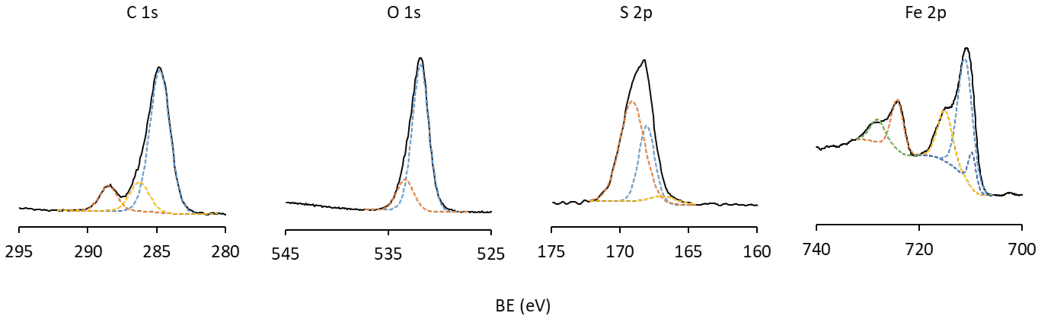

Figure S12 – Wide scan and high resolution XPS spectra of PAA brushes incubated in 10 mM $FeCl_2$ $4H_2O$

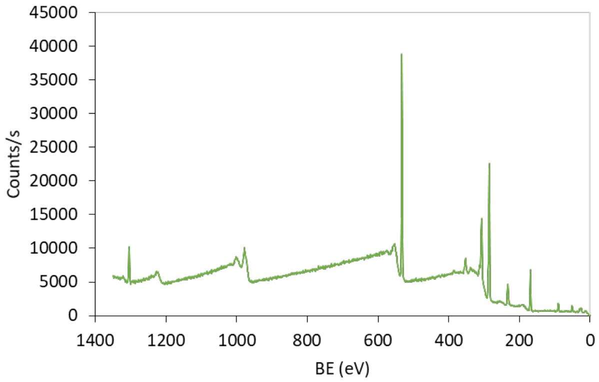

Name	Peak BE	Atomic %
C1s	285.1	48.05
O1s	532.14	29.8
S2p	168.54	7.05
S2s	232.37	6.29
Mg2s	89.78	3.01
Mg1s	1304.8	2.93
Mg2p	50.95	2.87

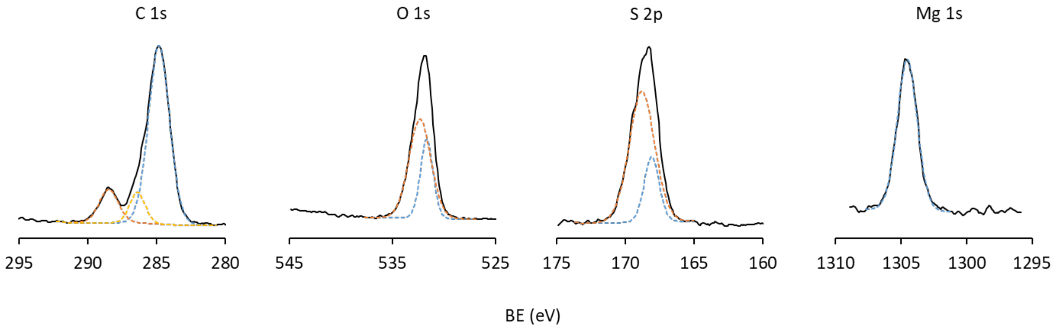

Figure S13 – Wide scan and high resolution XPS spectra of PAA brushes incubated in 10 mM $MgCl_2$

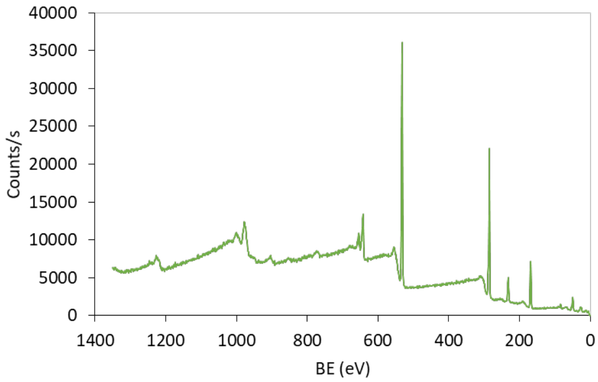

Name	Peak BE	Atomic %
C1s	285.02	49.89
O1s	532.04	30.19
S2p	168.36	7.3
S2s	232.18	6.4
Mn2p	642.44	3.3
Mn3p	49.16	2.92

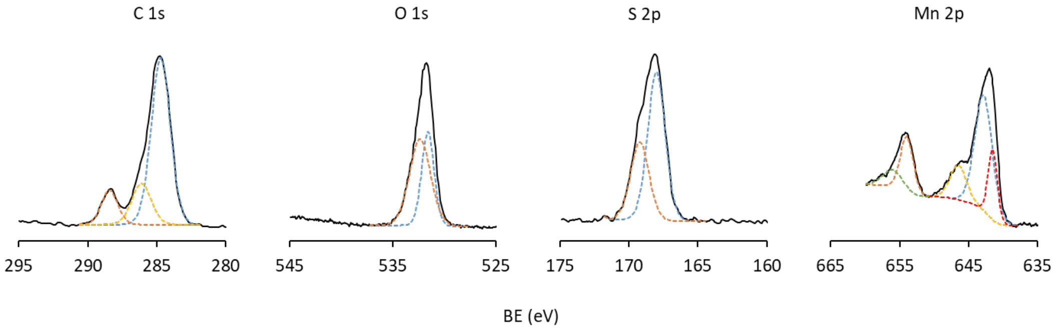

Figure S14 – Wide scan and high resolution XPS spectra of PAA brushes incubated in 10 mM $MnCl_2$ $4H_2O$

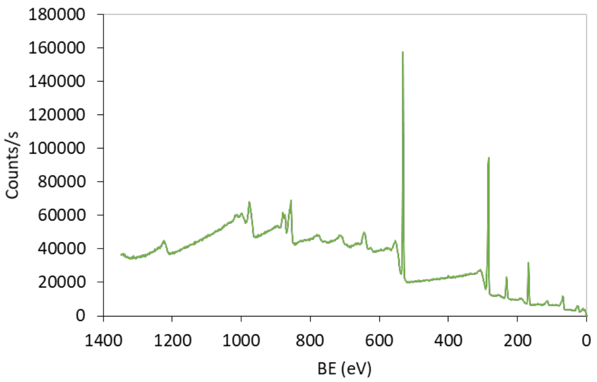

Name	Peak BE	Atomic %
C1s	284.46	44.13
O1s	531.33	27.27
Ni3p1	69.01	7.63
S2p	168.19	5.91
S2s	231.93	5.84
Ni2p1	877.01	3.65
Ni3s	113.98	3.41
Ni2p	856.39	2.15

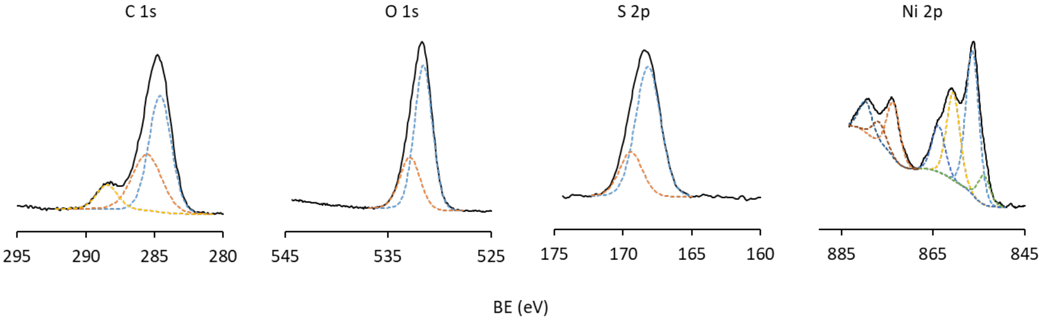

Figure S15 – Wide scan and high resolution XPS spectra of PAA brushes incubated in 10 mM $NiCl_2$

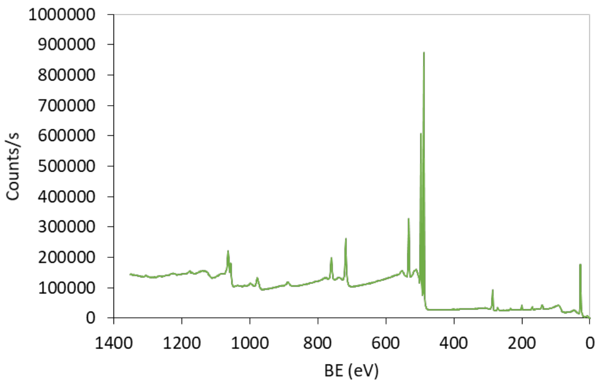

Name	Peak BE	Atomic %
O1s	531.32	23.98
C1s	285.08	18.79
Sn4s	139.15	10.48
Sn4d	26.45	10.2
Sn3d	487.05	10.12
Sn3p3	716.72	8.2
Sn3p1	758.49	7.89
Sn3s	886.36	5.9
Cl2p	199.22	1.88
S2p	168.81	1.48
S2s	232.29	1.08

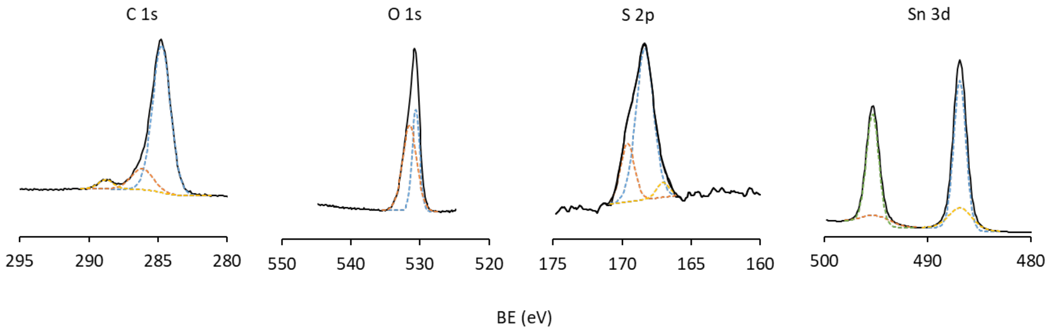

Figure S16 – Wide scan and high resolution XPS spectra of PAA brushes incubated in 10 mM $SnCl_2$

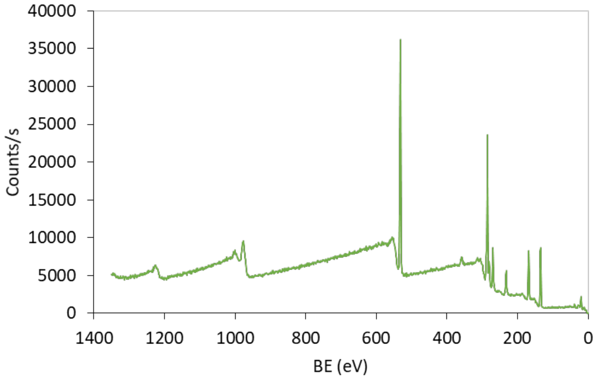

Name	Peak BE	Atomic %
C1s	285.11	43.52
O1s	532.01	30.17
S2s	232.15	7.69
S2p	168.29	7.34
Sr3s	358.34	3.67
Sr3d	134.55	2.82
Sr3p3	269.72	2.54
Sr4p	19.96	2.26

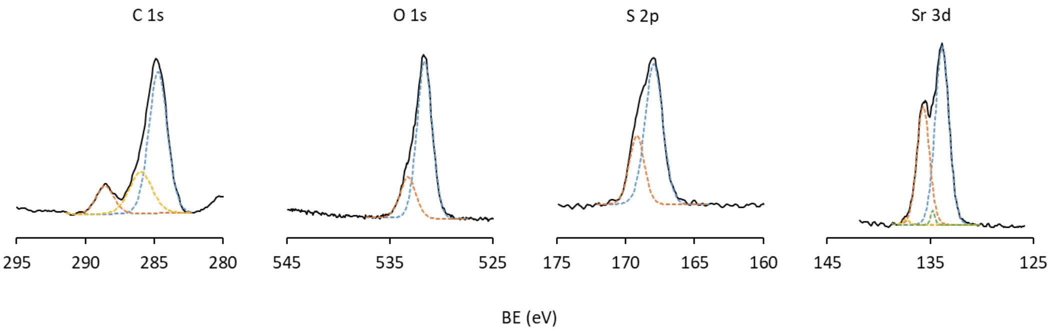

Figure S17 – Wide scan and high resolution XPS spectra of PAA brushes incubated in 10 mM $SrCl_2$ $6H_2O$

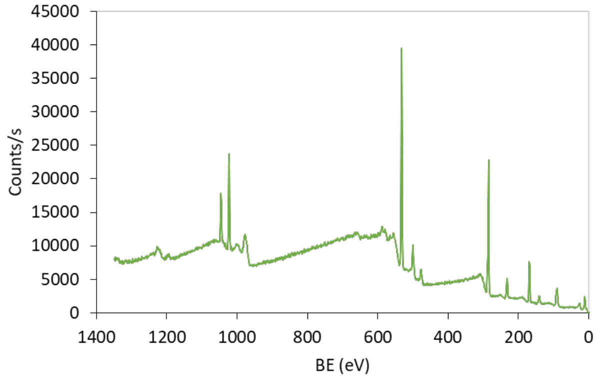

Name	Peak BE	Atomic %
C1s	285.14	45.68
O1s	532.24	27.67
S2p	168.89	6.67
S2s	232.7	5.79
Zn3s	140.91	4.61
Zn2p1	1045.9	3.36
Zn3p	90.36	3.3
Zn2p3	1022.86	2.93

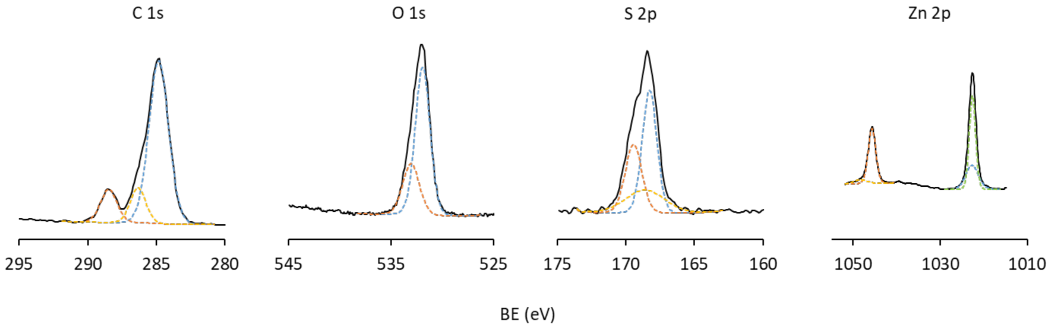

Figure S18 – Wide scan and high resolution XPS spectra of PAA brushes incubated in 10 mM $ZnCl_2$

S4. Additional info on swelling behaviour of PSPMA and PAA brushes

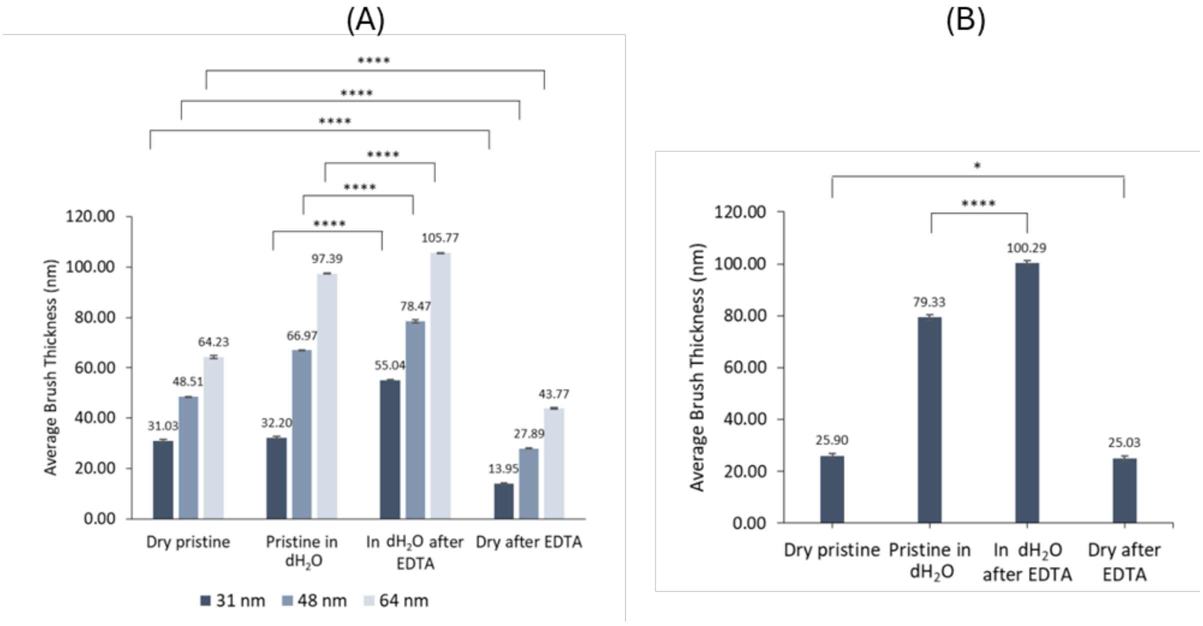

Figure S19 - Ellipsometry measurements of PSPMA (A) and PAA (B) brush samples with different initial dry thicknesses, tested dry or in deionised water, showing the impact of EDTA treatment on their swelling and collapsing behaviour (*: $p < 0.05$, ****: $p < 0.0001$).

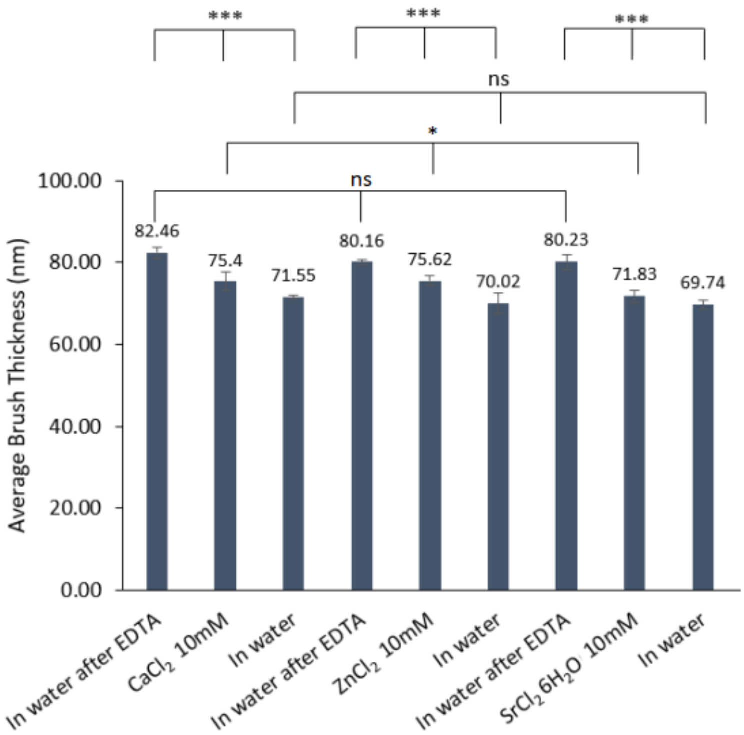

Figure S20 - *In-situ* ellipsometry measurements of PSPMA brushes showing the impact of residual cations trapped in the samples and their removal via EDTA treatment (ns: not statistically significant, *: $p \leq 0.05$, ***: $p \leq 0.001$).

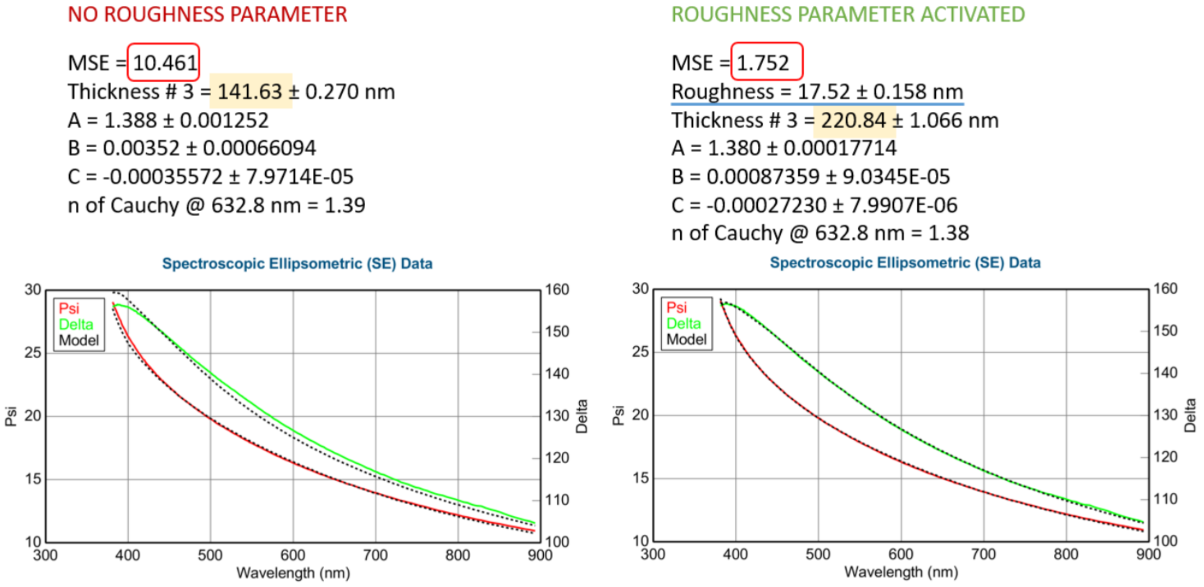

Figure S21 - Comparison of the Cauchy fitting of PSPMA brush sample submerged in a 10 mM MgCl2 aqueous solution, showing the difference in MSE and thickness when using no roughness parameter (left) or when activating the roughness parameter (right).

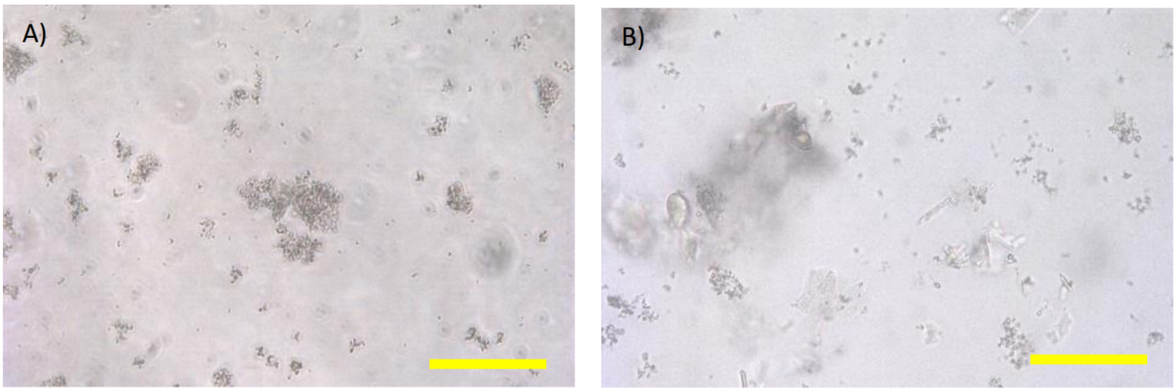

Figure S22 - Optical micrographs of a droplet of A) 10 mM $SnCl_2$ and B) 10 mM $ZnCl_2$ solutions showing sparse precipitate formation attributed to oxychloride crystals. Scale bar: 100 µm.

S5. Statistical tests

The **Tukey test** is a multiple comparison procedure and statistical test that compares every mean with every other mean to determine means that are significantly different from each other.

S5.1 Cations series

S5.1.1 PAA brushes

A one-way ANOVA test with Tukey's post hoc analysis was used to determine statistical significance of in-situ ellipsometry measurements of PAA brushes in various chloride salts containing different cations.

One way ANOVA

F	P value	P value summary
12.45	<0.0001	****

Turkey's test

Number of families	1
Number of comparisons per family	36
Alpha	0.05

Table S5 – Turkey's test results for PAA brushes in chloride salts of different cations.

Tukey's multiple comparisons test	Mean Diff,	Summary	Adjusted P Value
Sn vs. Mn	-0.139	ns	0.444
Sn vs. Zn	-0.141	ns	0.4241
Sn vs. Ca	-0.156	ns	0.2887
Sn vs. Cu	-0.238	*	0.012
Sn vs. Ni	-0.252	**	0.0061
Sn vs. Fe	-0.350	****	<0,0001
Sn vs. Sr	-0.418	****	<0,0001
Sn vs. Mg	-0.52	****	<0,0001
Mn vs. Zn	-0.002	ns	>0,9999
Mn vs. Ca	-0.017	ns	>0,9999
Mn vs. Cu	-0.099	ns	0.8344
Mn vs. Ni	-0.113	ns	0.7113
Mn vs. Fe	-0.211	*	0.0406
Mn vs. Sr	-0.279	**	0.0015
Mn vs. Mg	-0.381	****	<0,0001
Zn vs. Ca	-0.015	ns	>0,9999
Zn vs. Cu	-0.097	ns	0.8493
Zn vs. Ni	-0.111	ns	0.7306
Zn vs. Fe	-0.209	*	0.0442
Zn vs. Sr	-0.277	**	0.0017
Zn vs. Mg	-0.379	****	<0,0001
Ca vs. Cu	-0.082	ns	0.936
Ca vs. Ni	-0.096	ns	0.8565
Ca vs. Fe	-0.194	ns	0.0809

Ca vs. Sr	-0.262	**	0.0036
Ca vs. Mg	-0.364	****	<0,0001
Cu vs. Ni	-0.014	ns	>0,9999
Cu vs. Fe	-0.112	ns	0.721
Cu vs. Sr	-0.180	ns	0.1353
Cu vs. Mg	-0.282	**	0.0013
Ni vs. Fe	-0.098	ns	0.8419
Ni vs. Sr	-0.166	ns	0.2148
Ni vs. Mg	-0.268	**	0.0027
Fe vs. Sr	-0.068	ns	0.9786
Fe vs. Mg	-0.170	ns	0.1893
Sr vs. Mg	-0.102	ns	0.8106

S5.1.2 PSPMA brushes

A one-way ANOVA test with Tukey's post hoc analysis was used to determine statistical significance of in-situ ellipsometry measurements of PSPMA brushes in various chloride salts containing different cations.

<u>One way ANOVA</u>

F	P value	P value summary
77.75	<0.0001	****

<u>Turkey's test</u>

Number of families	1
Number of comparisons per family	36
Alpha	0.05

Table S6 – Turkey's test results for PSPMA brushes in chloride salts of different cations.

Tukey's multiple comparisons test	Mean Diff,	Summary	Adjusted P Value
Sn vs. Sr	-0.060	****	<0,0001
Sn vs. Zn	-0.062	****	<0,0001
Sn vs. Ca	-0.063	****	<0,0001
Sn vs. Mn	-0.091	****	<0,0001
Sn vs. Ni	-0.140	****	<0,0001
Sn vs. Mg	-0.152	****	<0,0001
Sn vs. Fe	-0.184	****	<0,0001
Sn vs. Cu	-0.188	****	<0,0001
Sr vs. Zn	-0.002	ns	>0,9999
Sr vs. Ca	-0.003	ns	>0,9999
Sr vs. Mn	-0.031	ns	0.0851
Sr vs. Ni	-0.080	****	<0,0001
Sr vs. Mg	-0.092	****	<0,0001
Sr vs. Fe	-0.124	****	<0,0001
Sr vs. Cu	-0.128	****	<0,0001
Zn vs. Ca	-0.001	ns	>0,9999
Zn vs. Mn	-0.029	ns	0.1342
Zn vs. Ni	-0.078	****	<0,0001
Zn vs. Mg	-0.090	****	<0,0001
Zn vs. Fe	-0.122	****	<0,0001
Zn vs. Cu	-0.126	****	<0,0001

Ca vs. Mn	-0.028	ns	0.1659
Ca vs. Ni	-0.077	****	<0,0001
Ca vs. Mg	-0.089	****	<0,0001
Ca vs. Fe	-0.121	****	<0,0001
Ca vs. Cu	-0.125	****	<0,0001
Mn vs. Ni	-0.049	***	0.0004
Mn vs. Mg	-0.061	****	<0,0001
Mn vs. Fe	-0.093	****	<0,0001
Mn vs. Cu	-0.097	****	<0,0001
Ni vs. Mg	-0.012	ns	0.9626
Ni vs. Fe	-0.044	**	0.002
Ni vs. Cu	-0.048	***	0.0005
Mg vs. Fe	-0.032	ns	0.0668
Mg vs. Cu	-0.036	*	0.0233
Fe vs. Cu	-0.004	ns	>0,9999

S5.2 Anions series

S5.2.1 PAA brushes

One-way ANOVA tests were performed to compare the effect of ionic strength and anion type on the swelling response of PAA brushes monitored via in-situ ellipsometry.

The statistical tests were performed to monitor the significant difference between the brush response to different anion types at same ionic strength or different ionic strengths of the same anion type.

Table S7 – One-way ANOVA test results for PAA brushes in different sodium salts solutions.

Variable parameter	Fixed parameter	F	P value	P value summary
Anions	0.01 M	11.35	0.003	**
Anions	0.05 M	17.82	0.0007	***
Anions	0.1 M	6.428	0.0159	*
Anions	0.5 M	17.46	0.0007	***
Anions	1 M	7.735	0.0095	**
Ionic strength	$NaClO_4$	27.04	<0.0001	****
Ionic strength	NaF	10.03	0.0016	**
Ionic strength	$NaNO_3$	1.232	0.3574	ns
Ionic strength	NaCl	4.653	0.0222	*

S5.2.2 PSPMA brushes

One-way ANOVA tests were performed to compare the effect of ionic strength and anion type on the swelling response of PAA brushes monitored via in-situ ellipsometry.

The statistical tests were performed to monitor the significant difference between the brush response to different anion types at same ionic strength or different ionic strengths of the same anion type.

Table S8 – One-way ANOVA test results for PSPMA brushes in different sodium salts solutions.

Lowest thickness (~40 nm)

Variable parameter	Fixed parameter	F	P value	P value summary
Anions	0.01 M	72.24	<0.0001	****
Anions	0.05 M	65.13	<0.0001	****
Anions	0.1 M	153.9	<0.0001	****
Anions	0.5 M	18.08	0.0006	***
Anions	1 M	19.63	0.0005	***
Ionic strength	$NaClO_4$	129	<0.0001	****

Ionic strength	$NaNO_3$	7.175	0.0054	**
Ionic strength	NaF	14.06	0.0004	***
Ionic strength	NaCl	14.06	0.0004	***

Medium thickness (~65 nm)

Variable parameter	Fixed parameter	F	P value	P value summary
Anions	0.01 M	92.97	<0.0001	****
Anions	0.05 M	156.4	<0.0001	****
Anions	0.1 M	154.6	<0.0001	****
Anions	0.5 M	279.2	<0.0001	****
Anions	1 M	201.7	<0.0001	****
Ionic strength	$NaClO_4$	143.3	<0.0001	****
Ionic strength	$NaNO_3$	128.5	<0.0001	****
Ionic strength	NaF	304.0	<0.0001	****
Ionic strength	NaCl	38.89	<0.0001	****

Higher thickness (~100 nm)

Variable parameter	Fixed parameter	F	P value	P value summary
Anions	0.01 M	42.15	<0.0001	****
Anions	0.05 M	5.337	0.026	*
Anions	0.1 M	1.433	0.3033	ns
Anions	0.5 M	6.172	0.0178	*
Anions	1 M	387.6	<0.0001	****
Ionic strength	$NaClO_4$	524.6	<0.0001	****
Ionic strength	$NaNO_3$	727.2	<0.0001	****
Ionic strength	NaF	60.76	<0.0001	****
Ionic strength	NaCl	9.318	0.0021	**
